# Fucosylated Human Milk Oligosaccharides and N-Glycans in the Milk of Chinese Mothers Regulate the Gut Microbiome of Their Breast-Fed Infants during Different Lactation Stages

**DOI:** 10.1128/mSystems.00206-18

**Published:** 2018-12-26

**Authors:** Yaqiang Bai, Jia Tao, Jiaorui Zhou, Qingjie Fan, Man Liu, Yuqi Hu, Yao Xu, Lilong Zhang, Jieli Yuan, Wenzhe Li, Xiaolei Ze, Patrice Malard, Zhimou Guo, Jingyu Yan, Ming Li

**Affiliations:** a College of Basic Medical Science, Dalian Medical University, Dalian, China; b Department of Gynaecology and Obstetrics, the First Affiliated Hospital of Jinzhou Medical University, Jinzhou, China; c Biostime (Guangzhou) Health Products Ltd., Guangzhou, China; d Dalian Institute of Chemical Physics, Chinese Academy of Sciences, Key Laboratory of Separation Science for Analytical Chemistry, Dalian, China; Pacific Northwest National Laboratory

**Keywords:** FUT2, HMOs, fucosylation, gut microbiome, milk N-glycans

## Abstract

The milk glycobiome has a significant impact on the gut microbiota of infants, which plays a pivotal role in health and development. Fucosylated human milk oligosaccharides (HMOs) and N-glycans on milk proteins are beneficial for the development of healthy gut microbiota, and the fucosylation levels of these glycans can be affected by the maternal fucosyltransferase 2 gene (*FUT2*). Here, we present results of longitudinal research on paired milk and stool samples from 56 Chinese mothers (CMs) and their breast-fed children. Changes of HMOs and fucosylated N-glycans in milk of CMs at different lactation stages were detected, which allowed characterization of the major differences in milk glycans and consequential effects on the gut microbiome of infants according to maternal *FUT2* status. Significant differences in the abundance of total and fucosylated HMOs between secretor and nonsecretor CMs were noted, especially during early lactation. Despite a tendency toward decreasing milk protein concentrations, the fucosylation levels of milk N-glycans increased during late lactation. The changes in the levels of fucosylated HMOs and milk N-glycans were highly correlated with the growth of *Bifidobacterium* spp. and *Lactobacillus* spp. in the gut of infants during early and later lactation, respectively. Enriched expression of genes encoding glycoside hydrolases, glycosyl transferases, ATP-binding cassette (ABC) transporters, and permeases in infants fed by secretor CMs contributed to the promotion of these bacteria in infants. Our data highlight the important role of fucosylated milk glycans in shaping the gut microbiome of infants and provide a solid foundation for development of “personalized” nutrition for Chinese infants.

**IMPORTANCE** Human milk glycans provide a broad range of carbon sources for gut microbes in infants. Levels of protein glycosylation in human milk vary during lactation and may also be affected by the stages of gestation and lactation and by the secretor status of the mother. This was the first study to evaluate systematically dynamic changes in human milk oligosaccharides and fucosylated N-glycans in the milk of Chinese mothers with different secretor statuses during 6 months of lactation. Given the unique single nucleotide polymorphism site (rs1047781, A385T) on the fucosyltransferase 2 gene among Chinese populations, our report provides a specific insight into the milk glycobiome of Chinese mothers, which may exert effects on the gut microbiota of infants that differ from findings from other study cohorts.

## Abstract (precis)

Human milk glycans provide a broad range of carbon sources for gut microbes in infants. Levels of protein glycosylation in human milk vary during lactation and may also be affected by the stages of gestation and lactation and by the secretor status of the mother. This was the first study to evaluate systematically dynamic changes in human milk oligosaccharides and fucosylated N-glycans in the milk of Chinese mothers with different secretor statuses during 6 months of lactation. Given the unique single nucleotide polymorphism site (rs1047781, A385T) on the fucosyltransferase 2 gene among Chinese populations, our report provides a specific insight into the milk glycobiome of Chinese mothers, which may exert effects on the gut microbiota of infants that differ from findings from other study cohorts.

## INTRODUCTION

Intestinal microbiota carry out functions that are vital for the health and development of newborns (1–4). Breastfeeding is one of the major factors guiding the establishment of gut microbiota in early life (5, 6). Human milk contains essential nutrients such as lactose, fatty acids, and proteins, as well as a constellation of bioactive compounds critical for the protection and appropriate development of the infant (7, 8). The milk glycobiome has a significant impact on shaping the gut microbiota of infants (9–11). Nondigestible sugars in breast milk, known as “human milk oligosaccharides” (HMOs), are protective to infants and function as prebiotics (12–14). Studies have shown that HMOs in breast milk are the main factors that induce the predominant development of bifidobacteria in the gut (15, 16). These bacteria in the intestine of breast-fed infants can utilize HMO components as carbon sources for their own growth. For example, α1,2-fucosylated HMOs have been shown to promote the growth of Bifidobacterium longum subsp., B. bifidum subsp., and B. breve spp., and these strains possess glycosyl hydrolase family 95 (GH95) or GH29 fucosidases that can hydrolyze 2′-fucosylated HMOs (16).

In addition to HMOs, large amounts of proteins in human milk are glycoproteins; they are utilized readily by infants and are also critical in the protection of newborns (17). Lactoferrin (LF) and immunoglobulins (Igs) are the most abundant glycoproteins in human milk, and they display broad antimicrobial and anti-inflammatory effects as well as many other biologic activities (18–20). LF and Igs in human milk contain highly fucosylated N-linked glycans in significantly higher proportions than are seen with N-glycans in bovine milk (21–24). Interestingly, certain microorganisms can cleave N-glycans from glycoproteins. Once released, these glycans can serve as carbon sources for gut microbes (25–27), which suggests that the glycosylation status of N-glycans in human milk also has a role in regulating the gut microbiome of infants.

The backbones of HMO and N-glycans are modified frequently by fucose and sialic acid to form various recognition motifs, such as blood-group antigens and Lewis antigens. Oligosaccharides can bind to other compounds in milk to form glycoconjugates (HMGs), which may have roles similar to those of HMOs (28). Among the enzymes that build HMGs in the mammary glands, fucosyltransferase 2 (FUT2) catalyzes the transfer of a fucose residue by means of an α1,2-linkage to the glycans found in human milk. *FUT2* is known as a “secretor” gene because of its role in expression of ABO blood groups in various secreted body fluids. Various mutations of *FUT2* that may inactivate its fucosyltransferase activity have been found among human populations (29). It has been reported that homozygotes for loss-of-function alleles in *FUT2* lack expression of ABH antigens in the gastrointestinal mucosa and bodily secretions and account for ∼20% of the world’s population (30, 31). The G428A mutation in *FUT2* at rs601338 is the most common polymorphism in Caucasian nonsecretors, and it has been found to associate closely with the gut *Bifidobacterium* spp. of breast-fed American infants (32). However, in Chinese populations, the mutation of G428A in *FUT2* is rare. Instead, the more common missense mutation of A385T at rs1047781 is responsible for dramatically decreased expression of ABH antigens (33, 34). However, until now, the degree to which the A385T single nucleotide polymorphism (SNP) of *FUT2* among Chinese mothers (CMs) affects expression of fucosylated HMOs and N-glycans in their milk and how these differences affect the gut microbiome of the offspring during lactation were not clear.

In this study, we undertook a longitudinal investigation into changes in the fucosylated HMOs and N-glycans that are abundant in the breast milk of secretor and nonsecretor CMs in northeastern China during 6 months of lactation. We also investigated the intestinal microbiota of infants fed solely by the breast milk of these CMs and evaluated the key phylotypes responsible for the differences between groups. In this way, we wished to (i) reveal the important role of fucosylated milk glycans in shaping the gut microbiome of infants and (ii) provide a solid foundation for further development of “personalized” nutrition for Chinese infants.

## RESULTS

### Distribution of secretor statuses among CMs.

Between May 2016 and December 2017, 56 infant/mother dyads in the First Affiliated Hospital of Jinzhou Medical University (Jinzhou, China) were selected to assess the effects of maternal milk glycome on the gut microbiota of infants during 6 months of lactation. All parents were born in North China, and the characteristics of the participants are summarized in Table 1.

**TABLE 1:** Subject characteristics

Characteristic	Values^*a*^	
	Secretors	Nonsecretors
Lactation day (no. of milk samples/no. of feces samples)		
6	43/43	13/13
42	35/39	10/10
120	25/24	6/7
180	19/21	5/6
Maternal age (yrs)	27.50 ± 0.521	29.00 ± 1.517
Maternal BMI^*b*^ (kg/m^2^)	25.89 ± 5.136	27.02 ± 3.115
Gestation duration (wk)	38.52 ± 1.333	39.45 ± 2.165
% of male infants (no. of males/total no. of infants)	41.86 (18/25)	46.15 (6/7)
Body weight of infants (kg) on lactation day:		
Birth	3.314 ± 0.094	3.191 ± 0.233
6	3.345 ± 0.087	3.394 ± 0.187
42	4.914 ± 0.159	5.033 ± 0.638
120	7.473 ± 0.231	7.600 ± 0.724
180	8.617 ± 0.307	9.167 ± 0.833

a Other than the sample numbers and the numbers of infants, data represent means ± standard deviations (SD). All of the infants were delivered vaginally and fed via breastfeeding.

^b^BMI, body mass index.

**FIG 1:**
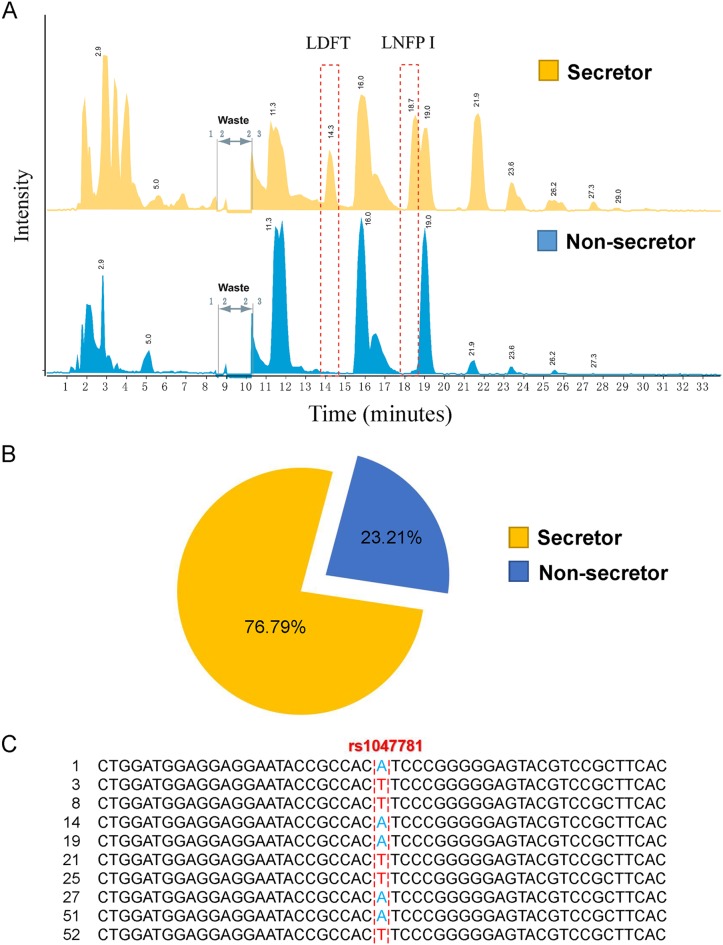
Distribution of secretor statuses among CMs. (A) Secretor gene status of CMs evaluated by the abundance of marker HMOs, including lactodifucotetraose (LDFT; *m*/*z* 669.2) and lacto-N-fucopentaose I (LNFP I; *m*/*z* 852.3) by MS. Cutoff values for the relative amounts of each marker were used to distinguish secretor women from nonsecretor women. (B) Proportions of secretor mothers and nonsecretor mothers enrolled in this study. A total of 13 nonsecretors were identified among 56 tested CMs. (C) Sequence examples of the partial *FUT2* gene amplicon containing the rs1047781 SNP (A→T) allele. A was mutated to T in the genome of the nonsecretor CMs.

Secretor status was examined by analyses of the oligosaccharides present in CMs’ milk using liquid chromatography coupled with mass spectrometry (LC-MS). This approach was adopted from a newly developed method based on a zwitterionic LC matrix with a mixed-mode action of hydrophilic interaction/cation exchange for cleanup and separation of HMOs (35). Neutral sugars were eluted, and acidic HMOs were resolved and identified. We used a different column material to allow acidic sugars to be eluted first and focused on detailed separation and neutral HMOs. Identification of HMOs was by electrospray ionization (ESI) MS, and initial assessment of secretor status was made by evaluation of markers in HMOs (32) (e.g., lactodifucotetraose [LDFT; *m*/*z* 669.2] and lacto-N-fucopentaose I [LNFP I; *m*/*z* 852.3]) (Fig. 1A).

Analyzing the relative abundances of these fucosylated HMOs, we identified 13 nonsecretors, which constituted 23.21% of the total CMs assessed (Fig. 1B). To investigate the correlation between different SNPs and secretor statuses among CMs, we tested the rs601338 and rs1047781 SNP sites in their genomic DNA by PCR and sequencing. None of the CMs exhibited SNP at rs601338, but the A→T mutation at rs1047781 was found in 13 of 56 CMs, who were identified as nonsecretors by LC-MS method (Fig. 1C; see also Table S1 in the supplemental material). This mutation may have affected *FUT2* expression, which can be predicted from the presence of 2′-fucosyallactose (2′-FL) and LNFP I in milk (36).

10.1128/mSystems.00206-18.7 TABLE S1 Sequencing of PCR amplicons harboring rs1047781/rs601338 mutation in nonsecretor samples. Download Table S1, DOCX file, 0.02 MB. Copyright © 2018 Bai et al. 2018 Bai et al. This content is distributed under the terms of the Creative Commons Attribution 4.0 International license.

### Dynamics of the major HMOs in the breast milk of CMs.

Using the LC-MS method described above, we carried out oligosaccharide profiling of the milk samples obtained from 56 CMs. Thirty oligosaccharide fractions (Table S2) were identified in milk samples. The top 16 major fractions that were more abundant than the other fractions were compared further between CMs with different secretor statuses (Table 2). For relative quantitation of isomeric oligosaccharides within mixed fractions resulting from incomplete separation, collision-induced dissociation–tandem MS (CID-MS/MS) was used. Unique fragment ions with *m*/*z* 325, 348, and 364 produced in negative-ion ESI-CID-MS/MS were characteristic of blood group H (α-1,2-fucosylgalactose), Lewis a, and Lewis x antigens, respectively (37). Hence, oligosaccharides with α1,2-linked fucose could be identified readily from the characteristic ions with *m*/*z* 325, and their abundances were reflected by the abundance of this unique fragment.

**TABLE 2:**
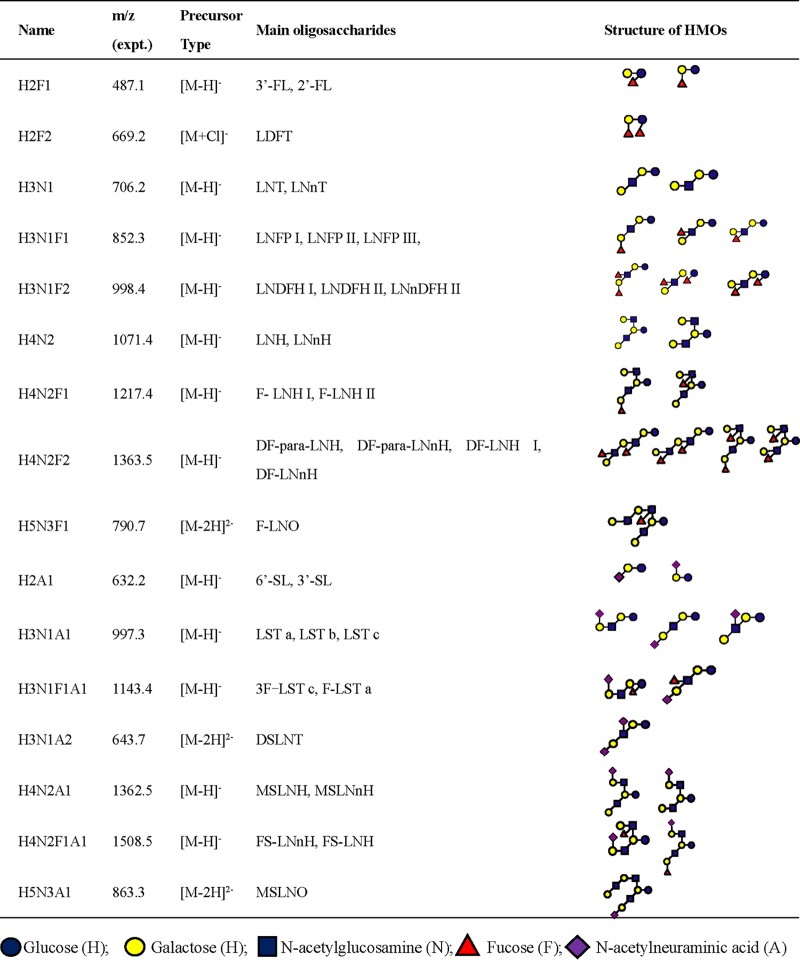
Composition and structures of the major HMO fractions found in milk of CMs

10.1128/mSystems.00206-18.8 TABLE S2 The major HMO fractions in breast milk detected by LC-MS. Download Table S2, DOCX file, 0.01 MB. Copyright © 2018 Bai et al. 2018 Bai et al. This content is distributed under the terms of the Creative Commons Attribution 4.0 International license.

**FIG 2:**
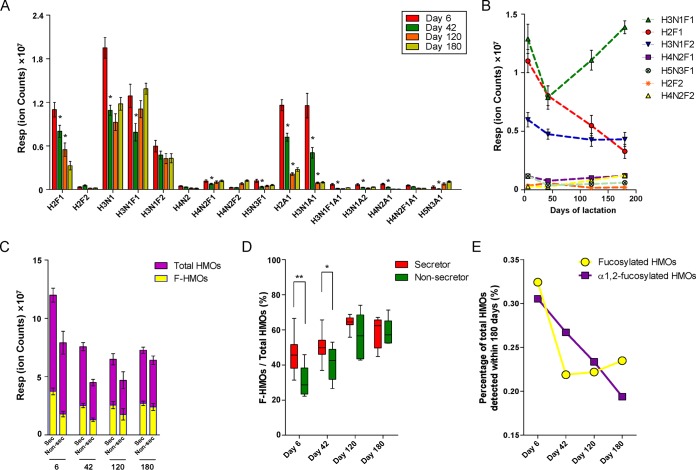
Dynamic of the major HMO fractions in breast milk of CMs. (A) The abundance of major HMO fractions detected in milk of CMs during 180 days of lactation. The asterisks (*) indicate that the abundance of the HMO group was significantly lower or higher than that measured at the earlier time point. Resp, response. (B) The dynamics of major fucosylated HMOs in milk of all the CMs during 180 days of lactation. (C) Comparison of the abundances of total and fucosylated HMOs (F-HMOs) between CMs with different secretor statuses. Sec, secretors; Non-sec, nonsecretors. (D) The ratio of fucosylated HMOs to total HMOs in milk of secretor and nonsecretors CMs. (E) The percentages of α1,2-fucosylated HMOs and total fucosylated HMOs in total HMOs detected at the four time points. The fragment ions (*m*/*z* 325) represent the α1,2-fucosylated HMOs extracted from the MS/MS spectra. Results of comparisons of the levels of the total HMOs and of the α1,2-fucosylated HMOs were normalized. Both the MS spectra and the MS/MS spectra were acquired in the negative-ion mode with an acquisition rate of 1 s per spectrum over ranges of *m*/*z* 300 to 2,000 (for MS) and *m*/*z* 50 to 2,000 (for MS/MS). Precursor-ion selection was performed automatically by the data system based on ion abundance. HMO identification and quantification were performed via the use of Agilent Mass Hunter Qualitative Analysis software (version B.03.01). All values are represented as means ± standard errors of the means (SEM). *, *P* < 0.05; **, *P* < 0.01 (two-tailed, unpaired Student's *t* test).

During 180 days of lactation, the abundance of most HMO groups in CM milk decreased gradually along with lactation progression (day 6 to day 42) (Fig. 2A) and did not change significantly at later stages (day 120 to day 180). However, some of the HMO groups, such as H3N1 and H3N1F1, showed an increasing pattern after 42 days (see Fig. S1A in the supplemental material). Also, significant reductions were observed (especially from day 6 to day 42) for most fucosylated HMOs (Fig. 2B). Comparing CMs with different secretor statuses, total and fucosylated HMOs were more abundant in the milk of secretor CMs than in that of nonsecretor CMs, especially during early lactation stages (Fig. 2C). The ratio of fucosylated HMOs to total HMOs in the milk of secretor CMs was significantly higher than in the milk of nonsecretor CMs by day 6 and day 42, but this difference decreased during the later stages of lactation (Fig. 2D; see also Fig. S1B). In our method, α1,2-fucosylated HMOs with characteristic α1,2-fucosylgalactose epitopes produced by FUT2 were also monitored by MS/MS. As shown in Fig. 2E, levels of α1,2-fucosylated HMOs were reduced along with lactation duration, but the total levels of fucosylated HMOs gradually stabilized in the late stage of lactation. The same decreasing tendency at day 6 to day 42 suggested that the *FUT2* mutation affected mainly the HMOs of CMs during the early stage of lactation.

10.1128/mSystems.00206-18.1 FIG S1 Dynamics of major HMO fractions during lactation. (A) The abundance of major HMO fractions during different lactation stages. (B) Comparison of the major 16 HMO fractions between secretor and nonsecretor CMs at the indicated time points during lactation. All values are represented as means ± SEM. *, *P* < 0.05; **, *P* < 0.01; ***, *P* < 0.001 (two-tailed, unpaired Student’s *t* test). Download FIG S1, TIF file, 0.7 MB. Copyright © 2018 Bai et al. 2018 Bai et al. This content is distributed under the terms of the Creative Commons Attribution 4.0 International license.

### Dynamics of the fucosylated N-glycans in the breast milk of CMs.

**FIG 3:**
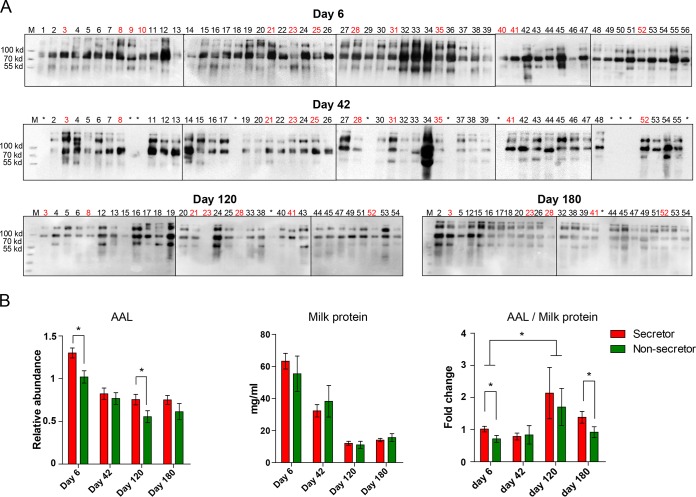
Dynamics of the fucosylated N-glycans in breast milk of CMs. (A) The fucosylation levels of milk glycoproteins during lactation detected by means of AAL blotting. Red numbers indicate the samples of nonsecretor CMs. Asterisks (*) indicate that no sample was collected by the indicated time point. (B) The fucosylation levels of milk proteins and the total protein concentrations were compared between secretor and nonsecretor groups. Density analysis of the AAL blot was performed using Quantity One software. All values are represented as means ± SEM. *, *P* < 0.05; **, *P* < 0.01 (unpaired Student's *t* test).

It has been found that N-glycans in human milk are highly fucosylated by glycosyltransferases such as FUT2, FUT3, and FUT8 (25). Therefore, we investigated if *FUT2* status also affects the fucosylation levels of milk glycoproteins by means of Aleuria aurantia lectin (AAL) blotting (which recognizes fucosylated glycan structures specifically) (38). An 80-kDa protein was highly fucosylated and was shown to be LF by Western blotting (Fig. 3A; see also Fig. S2A). The other major fucosylated milk proteins were found to be IgG and soluble IgA, data that are consistent with earlier studies (21–24). As expected, the fucosylation levels of milk proteins from secretor CMs were higher than those from nonsecretor CMs, although the total protein concentrations were comparable between groups (Fig. 3B; see also Fig. S2B). Notably, even though the protein concentrations reduced gradually as the HMOs did, the fucosylation levels of milk N-glycans increased during the late stage of lactation (day 120 and day 180) compared with those at day 6. A significant difference in the AAL/protein concentration ratios of secretor CMs and nonsecretor CMs was detected by day 180 (*P* = 0.0421).

10.1128/mSystems.00206-18.2 FIG S2 (A) Milk lactoferrin (LF) and immunoglobulins, including IgG and sIgA, were detected by Western blotting. (B) The protein samples stained by Coomassie brilliant blue (CBB). Download FIG S2, PDF file, 0.1 MB. Copyright © 2018 Bai et al. 2018 Bai et al. This content is distributed under the terms of the Creative Commons Attribution 4.0 International license.

### Milk microbiota of CMs with different secretor statuses.

**FIG 4:**
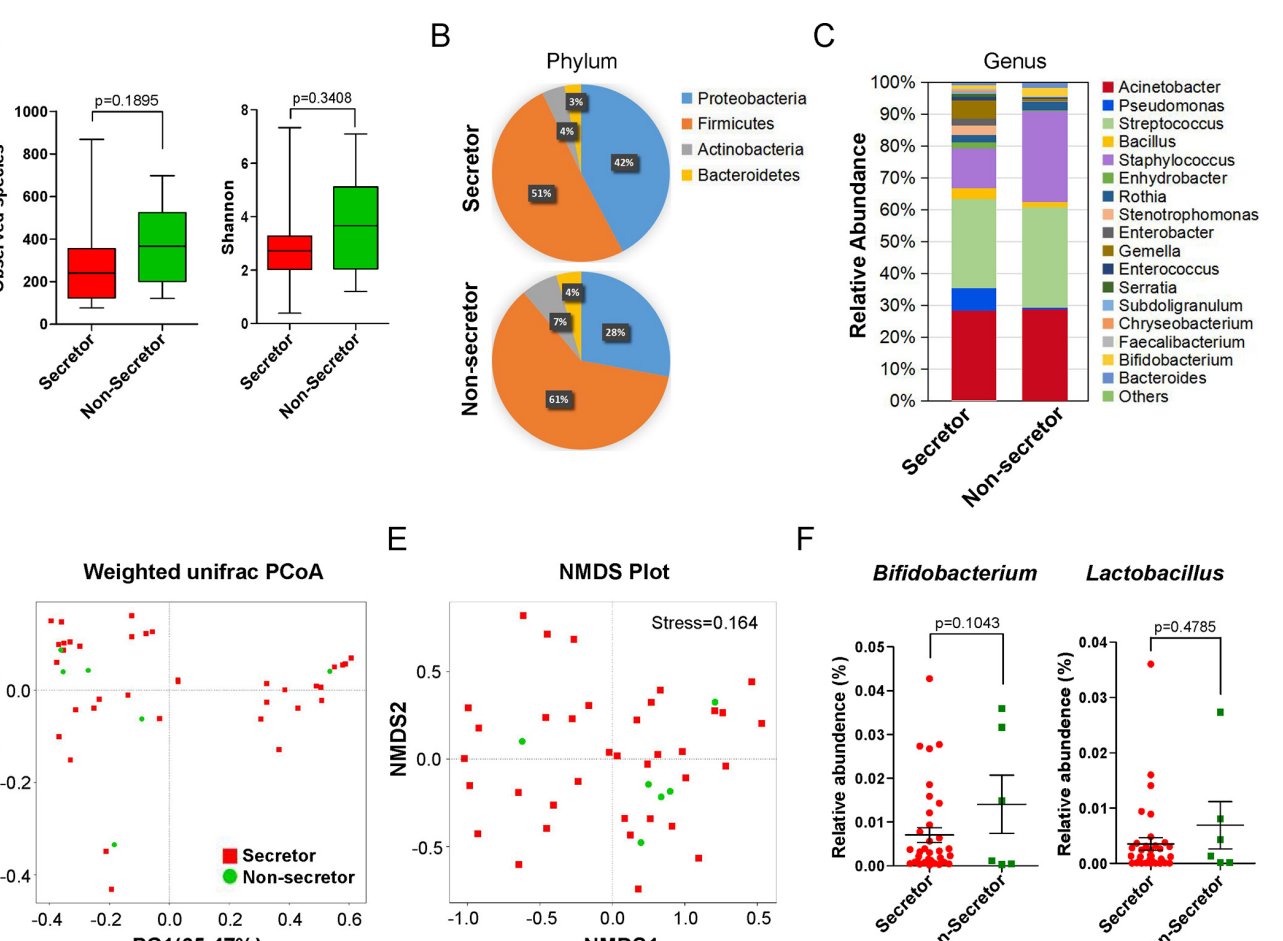
Milk microbiota of CMs with different secretor statuses. (A) The observed bacterial species and alpha diversity (indicated by Shannon index) in each group. (B) The predominant phyla detected in milk of secretor and nonsecretor CMs. (C) The relative abundances of milk bacterial genera, among which the major bacterial genera in the milk samples were *Acinetobacter* and *Streptococcus*. (D) The beta diversity of milk microbiota analyzed by principal-coordinate analysis (PCoA). (E) The beta diversity of milk microbiota analyzed by nonmetric multidimensional scaling (NMDS). (F) *t* test of the relative abundances of *Bifidobacterium* and *Lactobacillus* spp. between groups (*P* = 0.1043; *P* = 0.4785). All values are represented as means ± SEM [unpaired Student's *t* test]).

The human milk microbiota is one of the major factors that influences establishment of the gut microbiota of infants (39, 40). Therefore, we tested whether the milk microbiota of nonsecretor CMs was different from that of secretor CMs. 16S ribosomal DNA (rDNA) sequencing of the milk DNA of different CMs collected by day 6 revealed 280,245 high-quality filtered reads, which assembled into 66,725 effective tags per CM. These clean tags were clustered into 382 operational taxonomic units (OTUs) with 97% identity, which were classified further into “known” and “unclassified” bacterial groups (Fig. S3A). The two groups shared similar numbers of bacterial species and levels of alpha diversity (as indicated by the Shannon index values) (all *P* > 0.05) (Fig. 4A). The four predominant phyla detected in milk were *Firmicutes* (mean relative abundance, 56%), *Proteobacteria* (35%), *Actinobacteria* (5.5%), and *Bacteroidetes* (3.5%) (Fig. 4B). The abundance of *Firmicutes* in the milk of nonsecretor CMs was 10% higher than that of secretor CMs, whereas more *Proteobacteria* were found in the milk of secretor CMs. The major bacterial genera in these milk samples were *Acinetobacter* and *Streptococcus*, but their relative abundances were similar between groups (Fig. 4C). Beta diversity analyses by principal-coordinate analysis (PCoA) and nonmetric multidimensional scaling (NMDS) did not reveal a significant clustering pattern between these two groups (Fig. 4D and E). A Student's *t* test of the relative abundances of *Bifidobacterium* and *Lactobacillus* spp. showed no significant differences between groups (*P* > 0.05 for all) (Fig. 4F), which suggested that maternal secretor status had fewer effects on the microbial structure in milk.

10.1128/mSystems.00206-18.3 FIG S3 (A) OTU clustering and species annotation overview of milk microbiota. (B) OTU clustering and species annotation overview of gut microbiota of infants on day 6 postbirth. (C) OTU clustering and species annotation overview of gut microbiota of infants on day 42 postbirth. Download FIG S3, PDF file, 0.7 MB. Copyright © 2018 Bai et al. 2018 Bai et al. This content is distributed under the terms of the Creative Commons Attribution 4.0 International license.

### Gut microbiota of infants.

To investigate how the secretor status and milk glycans of CMs affected the gut microbiota of infants, we detected the fecal microbiota of infants at four time points (days 6, 42, 120, and 180 postbirth) during lactation by 16S rDNA sequencing. The fecal samples of infants collected by day 6 revealed 746,312 high-quality filtered reads, which assembled into 82,024 effective tags per infant. These clean tags were clustered into 177 OTUs with 97% identity, and representative sequences were used in the taxonomic analysis (Fig. S3B). By day 42, the sequencing reads were assembled into 82,400 effective tags per infant, which were clustered further into 147 OTUs (Fig. S3C), followed by a dramatic increase in OUT levels by day 120 (80,387 effective tags, 603 OTUs) and day 180 (78,213 effective tags, 1,014 OTUs), which suggested a time-dependent expansion of gut microbiota in infants. The OTUs were classified into 16 phyla, 40 classes, 71 orders, 134 families, 245 genera, and 98 species by day 180 data (taxonomic and phylogenetic information on these OTUs is provided in Table S3).

10.1128/mSystems.00206-18.9 TABLE S3 The total tags and OTU numbers derived from 16S rDNA sequencing of fecal samples of infants collected by day 120 postbirth. Download Table S3, DOCX file, 0.02 MB. Copyright © 2018 Bai et al. 2018 Bai et al. This content is distributed under the terms of the Creative Commons Attribution 4.0 International license.

**FIG 5:**
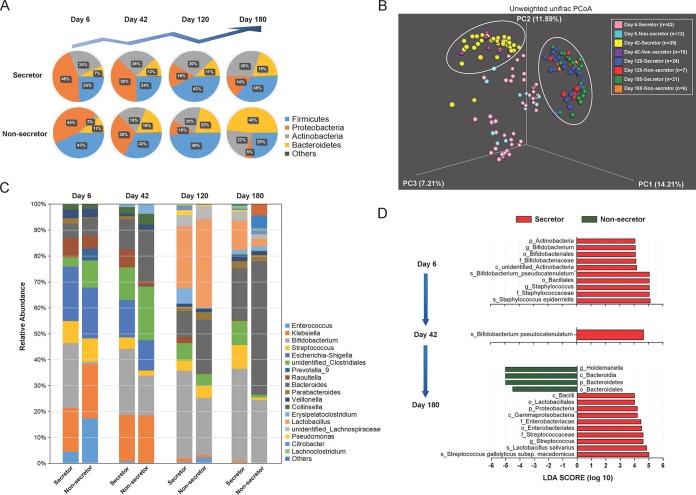
Gut microbiota of infants fed by CMs with different secretor statuses. (A) The dominant bacterial phyla in the gut of infants fed by secretor and nonsecretor CMs. (B) The beta diversity of gut microbiota in infants analyzed by PCoA during 180 days of lactation. PC1, PC2, and PC3 represent the top three principal coordinates that captured most of the diversity. The fraction of diversity captured by the coordinate is given as a percentage. (C) Changes in the average abundances of bacterial genera during lactation detected at the indicated time points. (D) The bacterial groups that showed significant differences between infants fed by secretor and nonsecretor CMs analyzed by the LEfSe (linear discriminant analysis effect size) method at different time points during lactation.

After systematic analyses, the observed species and Shannon index values (which reflected the alpha diversity of the gut microbiota of infants) showed no significant differences between the corresponding groups during 180 days of lactation (*P* > 0.05 for all). The dominant bacterial phyla in the gut of infants fed by secretor CMs underwent a gradual decrease in the number of *Proteobacteria* and a gradual increase in the number of *Firmicutes* and *Actinobacteria* (Fig. 5A), whereas the gut microbiota of infants fed by nonsecretor CMs fluctuated. By using PCoA, we decomposed all the sequencing data on bacterial genera into three factors that explained 33.01% of the variance (Fig. 5B; see also Fig. S4). All of these samples clustered mainly into three groups, which were highly correlated with the breastfeeding time. A significant difference in beta diversity between secretor and nonsecretor CMs by day 42 was detected by an unweighted UniFrac *t* test and other tests (Table S4). Increasing and decreasing patterns in the average abundances of the bacterial genera *Bifidobacterium*, *Enterococcus*, and *Klebsiella* were observed in both groups of infants (Fig. 5C). Notably, the abundance of *Bifidobacterium* in nonsecretor CM-fed infants was obviously lower than that in other infants throughout the lactation period. At the species level, there were four *Bifidobacterium* spp. detected in the gut of infants. B. pseudocatenulatum and B. breve were obviously more abundant than the others, and their abundances were significantly higher in infants fed by secretor mothers than in those fed by nonsecretors (Fig. S5). The linear discriminant analysis (LDA) effect size (LefSe) algorithm was adopted to identify the bacterial groups that showed significant differences in abundance between the two groups. Comparisons between groups revealed that the phylum of *Actinobacteria* was significantly more abundant in infants fed by secretor CMs than in other infants by day 6 (Fig. 5D). This result was contributed mainly by the abundance of B. pseudocatenulatum, a species that was also detected as the only biomarker in feces of secretor CM-fed infants collected by day 42. Interestingly, Staphylococcus epidermidis was also found to be significantly abundant in infants fed by secretor CMs. In contrast, no key phylotypes were detected in infants fed by nonsecretor CMs at day 6 and day 42. As the lactation duration increased, the species of bacteria that showed significant differences in abundance between the two groups changed, along with an alteration of major gut microbial groups. By day 120, no key phylotypes were detected in the two groups, suggesting a transition of gut microbial structure. By day 180, *Lactobacillales* and *Streptococcus* spp. became new biomarkers in the gut of infants fed by secretor CMs, and *Bacteroidetes* spp. were significantly more abundant in nonsecretor CM-fed infants.

10.1128/mSystems.00206-18.4 FIG S4 Comparisons of beta diversity (PCoA) of the gut microbiota of infants fed by secretor and nonsecretor CMs. Download FIG S4, PDF file, 0.9 MB. Copyright © 2018 Bai et al. 2018 Bai et al. This content is distributed under the terms of the Creative Commons Attribution 4.0 International license.

10.1128/mSystems.00206-18.5 FIG S5 (A) The abundance of microbes in the gut of infants at species level. (B) Comparison of the abundances of different *Bifidobacterium* spp. found in the gut of infants fed by secretor and nonsecretor CMs. All values are represented as means ± standard deviations (SD). *, *P* < 0.05; **, *P* < 0.01; ***, *P* < 0.001 (two-tailed, unpaired Student’s *t* test). Download FIG S5, TIF file, 1.8 MB. Copyright © 2018 Bai et al. 2018 Bai et al. This content is distributed under the terms of the Creative Commons Attribution 4.0 International license.

10.1128/mSystems.00206-18.10 TABLE S4 Statistical test of beta diversity levels of gut microbiota between infants fed by secretor mothers and nonsecretor mothers. Download Table S4, DOCX file, 0.01 MB. Copyright © 2018 Bai et al. 2018 Bai et al. This content is distributed under the terms of the Creative Commons Attribution 4.0 International license.

### Functional differences in gut microbial genomes in infants fed by secretor and nonsecretor CMs.

**FIG 6:**
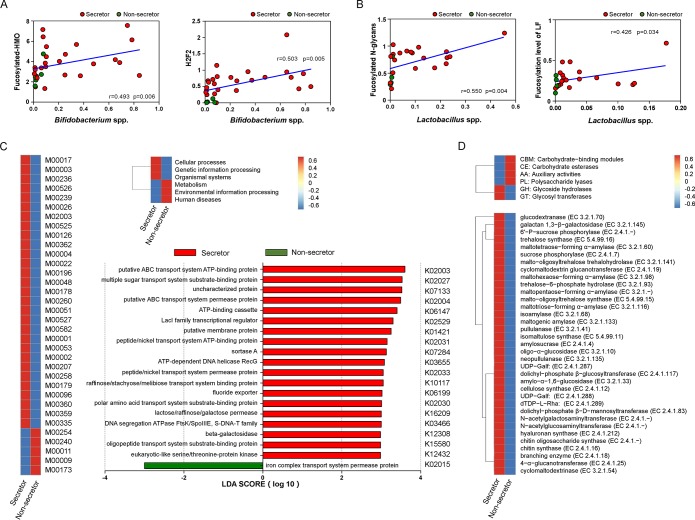
Functional differences in gut microbial genomes in infants fed by secretor and nonsecretor CMs. (A) The correlation between the level of total fucosylated HMOs/H2F2 and the abundance of *Bifidobacterium* spp. in the gut of infants at day 42 examined by Spearman’s rank analysis. (B) The correlation between fucosylated N-glycans/lactoferrin and abundance of *Lactobacillus* spp. in the gut of infants collected by day 180. (C) Heat map showing the major KEGG modules that were enriched in gut microbiota of infants fed by secretor or nonsecretor CMs. Thirty-four KEGG modules (left) were found differentially enriched in infants’ gut, and 29 in the secretor group were involved in cellular processes, genetic information processing, and organismal systems (top right). LEfSe was used to detect feature genes in each group, and LDA was performed to estimate the effect size of each feature gene (bottom right). (D) Heat maps showing the CAZy families (top) and enzymes (down) enriched in gut microbiota of infants fed by secretor or nonsecretor CMs.

Studies have reported that some bifidobacteria can utilize fucosylated HMO components efficiently to grow in the gut of infants (15–17). As expected, we found a positive correlation between the levels of total fucosylated HMOs and *Bifidobacterium* sp. abundance in the gut by day 6 and day 42 (Spearman’s rank analysis) (Fig. 6A; see also Fig. S6). A multiple-comparison test also revealed a significant correlation between H2F2 and *Bifidobacterium* spp. However, we did not find a positive correlation between the HMO levels and *Bifidobacterium* sp. abundance in the gut by day 120 or day 180. Instead, in evaluating the effects of fucosylated milk N-glycans, we found an obvious positive correlation between the levels of fucosylated N-glycans (especially LF) (Fig. 6B) and *Lactobacillus* spp. in samples from infants collected by day 180, which suggested a beneficial role of these N-glycans on *Lactobacillus* in the gut of infants during the later stage of lactation.

10.1128/mSystems.00206-18.6 FIG S6 Correlation between total fucosylated HMO levels and gut *Bifidobacterium* sp. abundance examined by Spearman’s rank analysis. Download FIG S6, TIF file, 1.9 MB. Copyright © 2018 Bai et al. 2018 Bai et al. This content is distributed under the terms of the Creative Commons Attribution 4.0 International license.

Using the Kyoto Encyclopedia of Genes and Genomes (KEGG) and Carbohydrate-Active Enzyme (CAZy) databases, we further evaluated the gut microbial functions of infants fed by secretor and nonsecretor CMs to identify the major enzyme/pathways involved in the metabolism of fucosylated HMOs and N-glycans. Thirty-four KEGG modules were found to be differentially enriched in the infant gut (Fig. 6C, left); 29 in the secretor group were involved in cellular processes, genetic-information processing, and organismal systems (Fig. 6C, top right). LEfSe analyses showed that beta-galactosidase (K12308) and many transport-system proteins, such as the ATP-binding cassette (ABC) transport system ATP-binding protein (K02003) and lactose/raffinose/galactose permease (K16209), were significantly abundant in the gut of infants fed by secretor CMs (Fig. 6C, bottom right). In contrast, only iron complex transport system permease protein (K02015) was enriched in infants fed by nonsecretor CMs. The CAZy database revealed that infants fed by secretor CMs were enriched mainly in genes associated with glycoside hydrolases and glycosyl transferases (Fig. 6D, top), including 35 abundant enzymes such as glucodextranase (EC 3.2.1.70) and galactan 1,3-β-galactosidase (EC 3.2.1.145) (Fig. 6D, bottom). Levels of enzymes such as N-acetylgalactosaminyltransferase (EC 2.4.1) and N-acetylglucosaminyltransferase (EC 2.4.1) were also enriched in the gut of these infants.

## DISCUSSION

In this report, we present results of longitudinal research performed on paired milk and stool samples from 56 CMs and their breast-fed children during 180 days of lactation. We detected changes in the abundances of HMOs and fucosylated N-glycans in the milk of CMs at different lactation stages. This information allowed us to characterize the major differences in milk glycans according to the secretor status of CMs as well as the consequential effects on the development of the gut microbiome of infants. We also compared the major enriched genes encoded by gut microorganisms whose levels might be driven by the activity of fucosylated milk glycans and which contribute to the establishment of a beneficial symbiotic relationship between gut microbiota and infants.

Glycans in human milk provide a broad range of carbon sources for gut microbes in infants. It was observed, not only in free glycans (41, 42), that the characteristics of protein glycosylation in human milk differ during lactation (21, 43) and may also be affected by gestation and lactation stages and by the mother’s secretor and Lewis blood group status (44). Changes in the content of 10 oligosaccharides in 446 milk samples from urban CMs were detected previously using high-performance liquid chromatography (HPLC) (45). That study provided an important and brief image of the major HMOs in the milk of CMs, but secretors and nonsecretors were not compared (45). The present study was the first to evaluate systematically the dynamics of HMOs and fucosylated N-glycans in the milk of CMs with different secretor statuses during 6 months of lactation. Given the unique SNP site on *FUT2* among Chinese populations, our report provides a specific insight into the milk glycobiome of CMs, which may exert effects on the gut microbiota of infants that are different from those seen with other study cohorts.

Several studies have shown the beneficial effects of HMOs on gut microbiota and immune development of infants, but detailed analyses of oligosaccharides remain very challenging due to the variety of structures and hydrophilicity (46). Recent advances in MS-based tools have provided a view of the HMO structures decorated with fucose and/or sialic acid moieties (35, 47, 48). In this study, we used LC-MS to profile HMOs, and data corresponding to the composition, structures, and relative amounts of HMOs were obtained employing 200 µl of human milk samples without derivatization or treatment. By this method, the secretor status of each lactating CM was also assigned according to her specific chromatogram.

Our results provide a horizontal comparison of 16 highly abundant HMO fractions in milk samples of CMs according to their ion responses in MS. In accordance with other studies, the richest content of HMOs was observed in colostrum, followed a sharp decrease after 42 days of lactation. We paid more attention to fucosylated HMOs due to their functions in affecting gut microbiota of infants. For most fucosylated HMOs, irrespective of secretor or nonsecretor status, a tendency of decreasing HMO concentrations and lactation durations was found. We investigated the variations in α1,2-fucosylated HMOs by analysis of their characteristic fragments during MS/MS. The decreasing abundance trend of α1,2-fucosylated HMOs suggested that FUT2 activity was reduced during lactation. This result was in accordance with the study by Thurl et al. (49). By their method, α1,2-fucosylated HMOs were calculated by the sum of the data from seven high-abundance HMOs; several other α1,2-fucosylated HMOs were ignored. However, in our method, data were calculated for all HMOs with α1,2-fucosylgalactose epitopes. Therefore, we provided a more clearly visible way to study the relationship between HMOs and fucosyltransferase.

Similarly to the levels observed for free glycans, milk protein glycosylation varies during lactation (21, 43). Our results showed a sharp decline in fucosylation levels of total milk proteins 42 days after birth compared with the level seen at 6 days. This result is in accordance with a lectin-based analysis of fucosylated glycoproteins of human skimmed milk during 47 days of lactation (50). However, during the later stages of lactation (days 120 and 180), the level of protein fucosylation was similar to that seen at day 42 based on the same volume of milk samples. This finding suggested that the glycosylation of human milk proteins is upregulated (because the protein concentration decreased largely along with lactation), and this may correlate with the different expression levels of glycosyltransferase during lactation (21). We also compared the fucosylation levels of milk glycoproteins in the different secretor groups. We found that the *FUT2* mutation mainly affected the fucosylation levels of milk glycoproteins during the early (day 6) stage of lactation (*P* = 0.0468) and that no significant change was detected by day 42, 120, or 120, although a lower abundance of fucosylated N-glycans was found in samples collected during these time points (but without significant differences) (*P* > 0.05 for all). One fact that we could not neglect was that the fucosylation of N-glycans in milk is modified not only by FUT2 but also by other fucosyltransferases, such as FUT3 and FUT8, which decorate the fucose residues by α1,3/4 or α1,6 linkages to bind on the N-glycans of various proteins (51). Also, the AAL-based detection of fucosylation levels is not able to distinguish different linkages of fucose. The α1,6-fucosylated glycotopes on milk glycoproteins, which are absent on HMOs (46), have been found to constitute an additional source of the ligand for lectin recognition (52). One study on the mammary glands of the tammar wallaby (*Macropus eugenii*) found that *FUT8* expression was upregulated in the later stage of lactation (53). That observation could support our finding that the fucosylation level of N-glycans in milk may increase after a long lactation regardless of the secretor status of CMs.

Gut microbiota of infants is essential for their health and development. This dynamic community is strongly affected by delivery mode and feeding patterns and other environmental factors (5). Recent studies (54, 55) have provided details of mother-to-child bacterial transmission during the first few months of life; however, after acquisition, the selection and stabilization of infant gut microbiota are dependent on multiple other factors, including the state of immune activation (3) and the carbon sources found in the infant gut (56). Our study cohort was composed of Chinese infants delivered by the vaginal route, and the infants were solely breast-fed during the study process; infants given antibiotics/probiotics or fed complementary foods due to a shortage of nutrients in CM milk were not included so that interference with gut microbiota could be ruled out. Therefore, the remaining major factors that might have affected the gut microbiome of infants were the maternal microbiome and glycans in milk.

The microbiota of milk has been revealed by several studies using culture-independent methods (57, 58). *Bifidobacterium* and *Lactobacillus* spp. are found in breast milk and can be transferred to the neonatal gut (59, 60), indicating that breastfeeding is a postnatal route for the mother-infant exchange of microbes. Factors such as geographic location and mode of delivery have been found to affect the diversity and abundance of breast milk microbiota (39). Interestingly, we did not find significant differences in the colostrum microbiota of CMs with different secretor statuses. This observation further highlights the effects of the milk glycobiome on the gut microbiota of infants, taking the secretor type of a CM as a major influencing factor.

Compared with the gut microbiota of infants fed by secretors, the gut microbiota of infants fed by nonsecretor CMs exhibited a very fluctuating pattern through 180 days of lactation. Similarly to what was seen in a study by Lewis et al. (32), bifidobacteria were established earlier and in greater quantities in secretor-fed infants than in those fed by nonsecretor CMs. The relative abundances of this genus continued to increase over 180 days of lactation in the secretor group. In contrast, a relatively lower abundance of bifidobacteria was found in nonsecretor CMs, although it also increased along with lactation duration. It has been reported that, even in children 2 to 3 years of age, the differences in the abundances of *Bifidobacterium* spp. in the gut are affected by the secretor status of the mother (61). We found that the predominant abundance of B. pseudocatenulatum was a major feature in the early (days 6 and 42) gut microbiota of infants fed by secretor CMs. This species has been found to exert several beneficial effects on infants (62, 63). However, it is not the most common Bifidobacterium species found in the feces of breast-fed infants (64); B. longum subsp. longum, B. longum subsp. infantis, and B. breve are more abundant. This phenomenon may due to the regional and ethnic differences between the participants in our study cohort and those in other cohorts. B. pseudocatenulatum can be shared by mothers and infants (65) and can also be detected in breast-fed infants (66). Our results suggest that B. pseudocatenulatum may be abundant in mothers living in northeastern China. On the other hand, the higher abundance of B. breve in infants fed by secretors than in those fed by nonsecretor CMs was inconsist with previous findings, as strains of B. breve have been shown to have the metabolic pathways of HMOs (64). In addition, we found a dominant presence of S. epidermidis in the gut of infants fed by secretor CMs (Fig. 5D). This finding is in accordance with an *in vitro* study (67) which showed that HMOs can promote the growth of staphylococci by causing greater utilization of amino acids in a medium. Thus, the high abundance of HMOs in the milk of secretor CMs may have contributed to the utilization of amino acids of S. epidermidis in the gut of infants. *Bacteroidetes* spp. are also glycan consumers (68), but their abundance in infants fed by nonsecretor CMs was significantly higher than that of secretor CMs by day 180. This result is in agreement with data from Smith-Brown et al. (61), which suggested that fucosylated glycans may not prefer these bacteria. Intriguingly, by the late stage of lactation (days 120 and 180), we found a pattern of increasing levels of *Lactobacillus* spp. in infants of both groups, and the order of *Lactobacillales* was the key phylotype that was significantly abundant in the secretor group.

Correlations between specific HMO structures in the milk and fecal microbiota of infants have been previously reported (60, 69). We undertook a correlation analysis based on our HMO classifications. Strongly positive correlations between total fucosylated HMOs/H2F2 and *Bifidobacterium* spp. were detected by days 6 and 42 postbirth of infants, suggesting an early promoting effect of fucosylated HMOs on gut bifidobacteria. This result is not surprising because genomic analyses of the bifidobacterial clade performed in the past decade (70, 71), with a particular focus on the genes involved in HMO degradation (72), have revealed that B. scardovii, B. infantis, and B. bifidum have an abundance of genes related to the degradation and transportation of host-derived glycans, including sialidases, fucosidase, hexosaminidases, β-galactosidase (73), ABC transporters (74), and permeases (75). Given that the linkages in milk glycans and glycoconjugates require these enzymes for cleavage, one would predict that functional analyses of the microbiome of breast-fed infants would show enrichment in these activities. Indeed, metagenomics of infant feces in the present study revealed significantly enriched expression of the genes encoding glycoside hydrolases and glycosyl transferases as well as of genes encoding many of the proteins and permeases involved in the ABC transport system.

We found that a pattern of increases in the levels of *Lactobacillus* spp. in the gut of infants at the late lactation stage correlated with increasing fucosylation levels of milk glycoproteins. LF is a natural glycoprotein that shows broad-spectrum antimicrobial activity, but growth-promoting effects of LF on specific *Lactobacillus* spp. have been reported (76, 77). High levels of fecal LF in neonates, particularly in the first days of life, could promote the abundance of *Lactobacillus* spp. and *Bifidobacterium* spp. (76). Molecular studies in bifidobacteria have suggested that the N-glycans on LF can be released by the activity of endo-N-acetylglucosaminidases (which belong to the GH18 family of glycoside hydrolases) and can serve as carbon sources for growth. The degraded N-glycans can be detected in the gut of breast-fed infants; such results are correlated with the abundance of *Bifidobacterium* spp. in the gut (66). However, until now, no scholars had characterized endo-N-acetylglucosaminidase-like enzymes from *Lactobacillus* spp. Nevertheless, analyses of the genomes of various species in this genus have indicated the existence of GH18 family glycoside hydrolases, such as the protein endo-N-acetylglucosaminidases (EHS83867.1) from Lactobacillus plantarum subsp. *plantarum* NC8 (NCBI GenBank [www.ncbi.nlm.nih.gov/protein/EHS83867.1]). No significant differences in fucosylation levels between secretor and nonsecretor groups were detected due to multiple fucosyltransferases being involved in decoration of milk N-glycans, which might have been upregulated during the later stages of lactation. However, the increasing abundances of *Lactobacillales* and *Bacteroidales* spp. in both groups indicated the hydrolyzing ability of these bacteria on fucoses joined by different linkages, as some studies have revealed the core fucose hydrolyzing abilities of these bacteria (78, 79). Therefore, further molecular mechanical studies regarding to the effects of milk protein N-glycans on these gut microbes are needed.

## MATERIALS AND METHODS

### Subjects and sample collection.

The study was approved by the ethical committees of Dalian Medical University and Jinzhou Medical University, China. A subset of 56 infant/mother dyads from the First Affiliated Hospital of Jinzhou Medical University (Jinzhou, China) were selected. Written informed consent was obtained from the parents before enrollment. Subjects were enrolled at approximately 34 weeks of gestation and asked to fill out detailed health history questionnaires. To eliminate the effect of the delivery mode on gut microbiome of infants, only those who gave birth by vaginal delivery were enrolled. The infants’ sex, weight, and gestational age at birth, as well as their diet throughout the study, were documented (Table 1).

Milk samples were collected in the morning on the indicated collection day postpartum using a modified published method (32). Subjects fully pumped the milk from one breast into a bottle, and the bottle was inverted six times, 10 ml of the bottle content was transferred into a 15-ml polypropylene tube, and the sample was subsequently frozen (−20°C) in the subjects’ kitchen freezers, transported within 1 week to the laboratory on dry ice, and stored at −80°C until processing.

Infant fecal samples were collected on the morning of each of the days when milk samples were collected. All of the infants consumed breast milk only, and infants who received antibiotics, probiotics, or formula powder because of diseases or lack of breast milk were excluded during the study period. Parents were instructed to immediately store the samples in −20°C until the time when the samples were transported on dry ice to the laboratory, where the samples were stored at −80°C before processing.

### Breast milk DNA extraction.

The genomic DNA of mother was extracted from breast milk using a Qiagen DNeasy blood and tissue kit (Qiagen, Venlo, Netherlands). Briefly, 2 ml of breast milk was spun in a microcentrifuge at 15,000 rpm for 30 min to pellet human cells. Cells were washed once in phosphate-buffered saline (PBS) and repelleted. The pellet was resuspended in 180 μl of PBS and incubated with 25 μl of proteinase K and 200 μl of buffer AL (a cell lysis buffer containing mainly guanidine hydrochloride and maleic acid; Qiagen, catalog no. 19075) for 10 min at 56°C. A 200-µl volume of ethanol was added to the sample and subjected to vortex mixing. The entire sample was loaded onto a spin column, and purification proceeded per the manufacturer’s recommended protocol from that point. DNA was eluted in 30 μl of elution buffer.

### FUT2 gene SNP detection.

Genomic DNA purified from each mother’s breast milk was amplified with primers FUT21-F (5′-CCTGGCAGAACTACCACCTG) and FUT21-R (5′-GGCTGCCTCTGGCTTAAAGA), which produced a 608-bp amplicon containing the rs601338 SNP (G→A) allele of the *FUT2* gene, and with primers FUT22-F (5′-CCTGGCAGAACTACCACCTG) and FUT22-R (5′-GGCTGCCTCTGGCTTAAAGA), which produced a 570-bp amplicon containing the rs1047781 SNP (A→T) allele of the *FUT2* gene. Each reaction mixture contained 10 μl of 2× GoTaq Green master mix (Promega, Madison, WI, USA), 1 μl of DNA, 1 μl of each primer (10 μM), and 7 μl of nuclease-free water. Cycling conditions were 95°C for 5 min followed by 35 cycles of 95°C for 30 s, 55°C for 30 s, and 72°C for 30 s. A final elongation was allowed at 72°C for 5 min. Successful amplification was confirmed by gel electrophoresis, and the PCR products were sent to Sangon Biotech Co. Ltd., Shanghai, China, for DNA sequencing.

### Extraction and detection of HMOs.

A 200-μl volume of milk was centrifuged at 9,000 rpm for 20 min at 4°C to remove lipid. Ethanol (400 μl) was then added to the skim milk before centrifugation was performed at 9,000 rpm for 10 min at 4°C to remove protein. The obtained supernatant was diluted 10-fold and used for analysis. The analysis of oligosaccharides was carried out on an LC-MS system, in which an Agilent 1290 series LC unit (35) was coupled with an Agilent 6540 series time-of-flight mass spectrometer. The drying gas temperature was 350°C, and the flow rate was 8.0 liters/min. Both MS and MS/MS spectra were acquired in the negative-ion mode with an acquisition rate of 1 s per spectrum over ranges of *m*/*z* 300 to 2,000 (for MS) and *m*/*z* 50 to 2,000 (for MS/MS). Precursor-ion selection was performed automatically by the data system based on ion abundance. Three precursors were selected from each MS spectrum to carry out product-ion scanning. The collision energy used for collision-induced dissociation (CID) was 30 V. HMO identification and quantification were performed via Agilent Mass Hunter Qualitative Analysis software (version B.03.01). A new column (ASP; 150 mm by 2.1 mm inside diameter [i.d.]) was used for the stationary phase. The mobile phase used for separation of the standard mixture was constituted of water and acetonitrile. The solvent gradient was performed at a flow rate of 0.2 ml/min as follows: 0 to 40 min, 20% to 50% water. The injection volume was 2 μl.

The mother’s secretor status phenotype in milk was determined by quantitating fucosylated glycan markers that have been previously described and assessed for sensitivity and specificity (35, 37). Secretor status was determined once per mother using milk samples collected on day 6 and markers that included lactodifucotetraose (LDFT; *m*/*z* 669.2) and lacto-N-fucopentaose I (LNFP I; *m*/*z* 852.3). Cutoff values for the relative amounts of each marker were used to distinguish secretor women from nonsecretors, as described previously (37). The fragment ions corresponding to *m*/*z* 325 were extracted from the MS/MS spectra for α1,2-fucosylated HMOs. The operation of normalization was used for data from comparisons of total HMOs and α1,2-fucosylated HMOs.

### Western and lectin blot analysis of milk N-glycans.

Each milk sample was defatted via centrifugation at 8,000 rpm for 10 min. After that, 1 μl of skimmed milk was taken and dissolved in 30 μl protein loading buffer (250 mM Tris-HCl, 0.5% bromophenol blue, 50% glycerol, 10% SDS, 5% beta- mercaptoethanol, pH 6.8) and then 10 μl of the sample mixture was subjected to sodium dodecyl sulfate-polyacrylamide gel electrophoresis (SDS-PAGE). After that, the proteins were transferred to polyvinylidene difluoride (PVDF) membranes at 300 mA for 50 min and were blocked for 1 h with 5% bovine serum albumin (BSA)–TBS-T (10 mM Tris-HCl, 150 mM NaCl, 0.1% Tween 20, pH 7.5) for Western blotting or lectin blotting. Following incubation with the appropriate primary antibodies (Abs) (human LF Abs were purchased from Abcam; IgG and IgA were purchased from Proteintech) or biotin-conjugated A.aurantia lectin (AAL) (Seikagaku, Tokyo, Japan) overnight, the membranes were washed twice with TBS-T. After washing, the blots were incubated with the corresponding secondary Abs conjugated with horseradish peroxidase (HRP) for AAL. Finally, specific proteins were visualized using an ECL system (Amersham). Density analysis was performed using Quantity One software.

### Determination of protein concentrations in milk.

Each milk sample was defatted via centrifugation at 8,000 rpm for 10 min. The middle layer of the solution was harvested, and the pH value of this solution was adjusted into 4.6 with HCl. The protein concentrations were quantified using a bicinchoninic acid (BCA) protein assay kit (TaKaRa), according to the manufacturer’s instructions.

### Fecal DNA extraction, PCR, and 16S rDNA amplicon data processing.

Microbial genome DNA was extracted from fecal samples of infants using an E.Z.N.A. isolation kit. A stool DNA kit (Omega Bio-tek, Inc.) was used according to the manufacturer's instructions. A Nanodrop 2000 spectrophotometer was used to evaluate the purity and concentration of isolated DNA. Universal primers (520F and 802R) were used to amplify the V4 region of 16S rDNA from metagenomic DNA in mice feces. Primer sets were modified with Illumina adapter regions for sequencing on the IlluminaGAIIx platform (Illumina, San Diego, CA, USA). The reverse primers were modified with an 8-bp Hamming error-correcting barcode to distinguish among samples. The 50-μl PCR mixture contained the following components: 100 ng of DNA template, 5 μl PCR buffer, 1-μl volumes of deoxynucleoside triphosphates (dNTPs), 0.25 μl HotStarTaq Plus DNA polymerase (Qiagen), and 2.5 pmol of each primer. The PCR program consisted of an initial step at 95°C for 5 min; 30 cycles of 94°C for 45 s, 55°C for 45 s, and 72°C for 60 s; and a final extension at 72°C for 8 min. PCR products were checked by 1.5% (wt/vol) agarose gel electrophoresis in 0.5 mg/ml ethidium bromide and purified with a QIAquick gel extraction kit (Qiagen). Sequences of 16S rDNA were detected by the use of an Illumina HiSeq system (Novogene Bioinformatics Technology Co. Ltd., Beijing, China) (reconstructed cDNA sequence, 2 × 250 bp). The sequences obtained after quality control analysis were used in the present analysis and were uploaded to QIIME (Quantitative Insights Into Microbial Ecology, v1.8.0) for further study. The operational taxonomy units (OTUs) of representative sequences at a similarity cutoff of 97% and their relative (alpha diversity) abundances were used to calculate Shannon index values and other index values by UCLUST. The abundance and diversity of the OTUs (beta diversity) were examined using principal-coordinate analysis (PCoA) and nonmetric multidimensional scaling (NMDS) with unweighted UniFrac analysis in R software. The statistical significance of the separation among groups was assessed by the linear discriminant analysis effect size (LEfSe) method based on linear discriminant analysis scores exploited by Curtis Huttenhower (http://huttenhower.sph.harvard.edu/galaxy/), which used the nonparametric factorial Kruskal-Wallis and Wilcoxon rank sum tests to identify key OTUs for separating different treatment groups at a significance level of 0.05. This work was conducted by Levelgene Bio-Pharm Technology Co., Ltd., Dalian, China.

### Metagenomic sequencing and gene catalogue construction.

The qualified DNA samples taken from infant feces samples on day 42 postbirth were randomly broken into fragments of about 350 bp by the use of a Covaris sonicator, and the whole library was prepared by terminal repair, A tail processing, sequencing ligation, purification, and PCR amplification. After the library was constructed, preliminary quantification was performed using Qubit 2.0, and the each sample in the library was diluted to 2 ng/µl. Then, the insertion size each sample in the library was detected using an Agilent 2100 system. After the insertion was confirmed to be of the expected size, the effective concentrations of the samples in the library were determined by quantitative PCR (qPCR). Accurate quantification (effective library concentration of >3 nM) was confirmed to ensure library quality. After the library was qualified, the different libraries were pooled according to the effective concentrations and the target data volumes and sequenced by the use of an Illumina HiSeq system (Novogene Bioinformatics Technology Co. Ltd., Beijing, China).

Raw data obtained by sequencing have a certain proportion of low-quality data. In order to ensure the accuracy and reliability of the subsequent information analysis results, the raw data should first be subjected to quality control and host filtration as follows. (i) Remove the low-quality bases (mass value of ≤38) that exceed a certain ratio (default, 40 bp) of reads. (ii) Remove N bases for a certain proportion of reads (default, 10 bp). (iii) Remove the overlap reads between the adapters if the base pairs exceed a certain threshold (default, 15 bp). (iv) If a sample is host contaminated, compare it with entries in the host database. The filtering may be from the host (the default is SOAPaligner software; parameter setting: identity ≥ 90%, -l 30, - v 7, -M 4, -m 200, -x 400) reads; get valid data (Clean Data). Starting from each sample and mixed assembled Scaftigs (continuous sequences formed by multiple initial contigs lined up in a scaffold with putative sequence overlaps; ≥500 bp), open reading frame (ORF) prediction was performed using MetaGeneMark, and the information with a length of less than 100 nucleotides (nt) was filtered out of the prediction results. Redundancies in the ORF prediction results for each sample and mixed assembly were removed using CD-HIT software to obtain a nonredundant initial gene catalogue, which was clustered by the use of an identity value of 95%, with coverage of 90% by default, and the longest sequence was selected. For representative sequences, the parameters are as follows: -c 0.95, -G 0, -aS 0.9, -g 1, -d 0. The genes supporting ≤2 reads in each sample were filtered out to obtain the gene catalogue (Unigenes), which was finally used for subsequent analysis. Alignment of Unigenes with sequences of bacteria, fungi, archaea, and viruses from the NCBI NR database (Version: 2016-11-05) was performed using DIAMOND software (blastp) (E value, ≤1e−5). For the comparisons performed with each sequence, the result of comparisons of E value less than or equal to the minimum E value * 10 is selected for subsequent analysis. After filtering, since each sequence may have a number of alignment results, multiple examples of classification information for different species are obtained. In order to ensure the biological significance of the data, the LCA algorithm (system classification applied to MEGAN software) is adopted, and the first branch subsequently appears.

### Statistical analysis.

Statistical analysis of results of comparisons between secretor and nonsecretor groups was performed using an unpaired, two-tailed *t* test with Welch’s correction and Graph Pad Prism Version 5. *P* values of <0.05 were considered statistically significant (*, *P* < 0.05; **, *P* < 0.01; ***, *P* < 0.001). The Shannon index at the genus level was calculated with QIIME (Version 1.7.0). With a normalized relative abundance matrix, LEfSe was used with the Kruskal-Wallis rank sum test to detect features with significantly different abundances between assigned taxa and LDA was performed to estimate the effect size of each feature. A significance alpha of 0.05 and an effect size threshold of 3 were used for the biomarkers discussed in this study. Data correlating to differential abundances of genes and genera and knockout (KO) modules were tested by Wilcoxon rank sum test, and *P* values were corrected for multiple testing with the Benjamin & Hochberg method. Only genera for which the average relative abundance value was ≥10^−4^ and that were found in at least six subjects were considered in the analyses. Correlations between milk glycans and gut microbiota (the relative abundances of bacterial genera) were analyzed by calculating Spearman’s rank correlation coefficients by the assistance of Graphpad Prism Version 5. Only relationships having an absolute Spearman’s correlation value above 0.3 with a *P* value of less than 0.05 were selected.
