# Supplementary material for: Fucosylated Human Milk Oligosaccharides and N-Glycans in the Milk of Chinese Mothers Regulate the Gut Microbiome of Their Breast-Fed Infants during Different Lactation Stages
Source: mSystems. 2018 Dec 26;3(6):e00206-18. doi: 10.1128/mSystems.00206-18 (PMC6306508; doi:10.1128/mSystems.00206-18)
Supplement: TABLE S1 [file sys006182308st1.docx]

TABLE S1. *Sequencing of PCR amplicons harboring* rs1047781/rs601338 mutation in non-secretor samples

| Sample number | rs1047781 | rs601338 |
| --- | --- | --- |
| 3 | TATGTGCTCAGGACTTCAGAATTATACGTTTCTCTTGATATTGGCTTATGGGCGCATTCCTGTCTTTTATTTATTTCTCA  TGCCACGCGGGCGGCTCTGACGTCCAGACAGCGATGCCATGGTGTGATATCGGCTCAACGCCGTCTCCATTTCCATGGGTTCAATTAATTGGCCTGGCTGGCCCTCCCCAGTAGTTGGGACTACTCGTGACGCTCACCACGCCCAATCAATCTTTATACTTTTGGTGGCGGTTGGTTTCACCCGAGATCACCCCAACGGACTCCATCCCGTGACACCGAGACCCACCCCCCCCCACCACAAATAATGTAAAAATGACAATACTTACCCACTGAACCCTCCCTTCACTTGACGATTCTGAAATCATAACCCTGCGGAAAATATTTTGACATATACACGGATGTGTGGTGTCTTATA | ACCGCCACTTCCCGGGGGAGTACGTCCGCTTCACCGGCTACCCCTGCTCCTGGACCTTCTACCACCACCTCCGCCAGGAGATCCTCCAGGAGTTCACCCTGCACGACCACGTGCGGGAGGAGGCCCAGAAGTTCCTGCGGGGCCTGCAGGTGAACGGGAGCCGGCCGGGCACCTTTGTAGGGGTCCATGTTCGCCGAGGGGACTATGTCCATGTCATGCCAAAAGTGTGGAAGGGGGTGGTGGCCGACCGGCGATACCTACAGCAGGCCCTGGACTGGTTCCGAGCTCGCTACAGCTCCCTCATCTTCGTGGTCACCAGTAATGGCATGGCCTGGTGTCGGGAGAACATTGACACCTCCCACGGTGATGTGGTGTTTGCTGGCGATGGCATTGAGGGCTCACCTGCCAAAGATTTTGCTCTACTCACACAGTGTAACCACACCATCATGACCATTGGGACGTTCGGGATCTGGGCCGCATACCTCACGGGCGGAGACACCATCTACCTGGCCAATTACACCCTCCCCGACTCCCCTTTCCTCAAAATCTTTAAGCCAGAGGCAGCCAAAA |
| 8 | CAAGCACTCACTGGTTAGGACTTCAGAATCATCGTTTCTCTTGATATTGACTTATGGGCGTATTCCTGTCTTCCCATTTATTTCTCTTGCCGAGGCAGGCGGCTCTGTCGTCCAGAGAGGAGTGCCATCCGGGCCGATCGGCTCAACCCCGTCTCCATTACAATGGGTTCAATTAATTGGCCTGGCTGACCCTCACCAGTAGTTGGGACTACTCGGGACGCTCACCACGCCCAATCAATCTTTATACTTTTGGTGGCGGTTGGTTGACCCGAGATCACCCCGACGGACTCCATCCCGTGACACCGAGCCCCACCCCCCCCCGCCACACATAAATTTGGGAAATAAATACTGACCCACTGAACCCGGCCTACTCTTGACGATTCTGATATCATAACCCTGTGGAAAATCTTTTGACATATACAGGGATGTGTGGTGTCGTGT | CCGCCACTTCCCGGGGGAGTACGTCCGCTTCACCGGCTACCCCTGCTCCTGGACCTTCTACCACCACCTCCGCCAGGAGATCCTCCAGGAGTTCACCCTGCACGACCACGTGCGGGAGGAGGCCCAGAAGTTCCTGCGGGGCCTGCAGGTGAACGGGAGCCGGCCGGGCACCTTTGTAGGGGTCCATGTTCGCCGAGGGGACTATGTCCATGTCATGCCAAAAGTGTGGAAGGGGGTGGTGGCCGACCGGCGATACCTACAGCAGGCCCTGGACTGGTTCCGAGCTCGCTACAGCTCCCTCATCTTCGTGGTCACCAGTAATGGCATGGCCTGGTGTCGGGAGAACATTGACACCTCCCACGGTGATGTGGTGTTTGCTGGCGATGGCATTGAGGGCTCACCTGCCAAAGATTTTGCTCTACTCACACAGTGTAACCACACCATCATGACCATTGGGACGTTCGGGATCTGGGCCGCATACCTCACGGGCGGAGACACCATCTACCTGGCCAATTACACCCTCCCCGACTCCCCTTTCCTCAAAATCTTTAAGCCAGAGGCAGCCAAGA |
| 9 | CAAGCTCTTACTGGTCAGAACTTCAGAATTATACGTTTCTCTTGATATTTACTTATGGGCTCATTTCTGTTTTCCCATTTATTTCTCTTGAGAGGCGTCGGCTCTGACGTCCAGACAGGAGTGCCATCCGGTGATCTCGGCTCAACGCCGCCGCCACCTCCCTGGGTTCAAGCGATTGGCCTGGCTGACCCTCCCCAGTAGTTGGAACTACTCGTGACGCTCACCACGCCCAATCAATCTTTATACTTTTGGTGGCGGTTGGTTTCACCCGAGATCACCCCGACGGACTCCATCCCGTGACACCGAGACCCACCCCCCCCCGCCACACATAATGTAAAAATGACAATACTGACCCACTGAACCCTGCCTACACTTGACGATTCTGATATCATAACCCTGCGGAAAATATTTTGACATATACACGGAGATGTGGTGTCTTATA | CCGCCACTTCCCGGGGGAGTACGTCCGCTTCACCGGCTACCCCTGCTCCTGGACCTTCTACCACCACCTCCGCCAGGAGATCCTCCAGGAGTTCACCCTGCACGACCACGTGCGGGAGGAGGCCCAGAAGTTCCTGCGGGGCCTGCAGGTGAACGGGAGCCGGCCGGGCACCTTTGTAGGGGTCCATGTTCGCCGAGGGGACTATGTCCATGTCATGCCAAAAGTGTGGAAGGGGGTGGTGGCCGACCGGCGATACCTACAGCAGGCCCTGGACTGGTTCCGAGCTCGCTACAGCTCCCTCATCTTCGTGGTCACCAGTAATGGCATGGCCTGGTGTCGGGAGAACATTGACACCTCCCACGGTGATGTGGTGTTTGCTGGCGATGGCATTGAGGGCTCACCTGCCAAAGATTTTGCTCTACTCACACAGTGTAACCACACCATCATGACCATTGGGACGTTCGGGATCTGGGCCGCATACCTCACGGGCGGAGACACCATCTACCTGGCCAATTACACCCTCCCCGACTCCCCTTTCCTCAAAATCTTTAAGCCAGAGGCAGCCAAA |
| 10 | CAAGCACTCACTGGTTAGGACTTCAGAATCATCGTTTCTCTTGATATTGACTTATGGGCGTATTCCTGTCTTCCCATTTATTTCTCTTGCCGAGGCAGGCGGCTCTGTCGTCCAGAGAGGAGTGCCATCCGGGCCGATCGGCTCAACCCCGTCTCCATTACAATGGGTTCAATTAATTGGCCTGGCTGACCCTCACCAGTAGTTGGGACTACTCGGGACGCTCACCACGCCCAATCAATCTTTATACTTTTGGTGGCGGTTGGTTGACCCGAGATCACCCCGACGGACTCCATCCCGTGACACCGAGCCCCACCCCCCCCCGCCACACATAAATTTGGGAAATAAATACTGACCCACTGAACCCGGCCTACTCTTGACGATTCTGATATCATAACCCTGTGGAAAATCTTTTGACATATACAGGGATGTGTGGTGTCGTGT | ACCGCCACTTCCCGGGGGAGTACGTCCGCTTCACCGGCTACCCCTGCTCCTGGACCTTCTACCACCACCTCCGCCAGGAGATCCTCCAGGAGTTCACCCTGCACGACCACGTGCGGGAGGAGGCCCAGAAGTTCCTGCGGGGCCTGCAGGTGAACGGGAGCCGGCCGGGCACCTTTGTAGGGGTCCATGTTCGCCGAGGGGACTATGTCCATGTCATGCCAAAAGTGTGGAAGGGGGTGGTGGCCGACCGGCGATACCTACAGCAGGCCCTGGACTGGTTCCGAGCTCGCTACAGCTCCCTCATCTTCGTGGTCACCAGTAATGGCATGGCCTGGTGTCGGGAGAACATTGACACCTCCCACGGTGATGTGGTGTTTGCTGGCGATGGCATTGAGGGCTCACCTGCCAAAGATTTTGCTCTACTCACACAGTGTAACCACACCATCATGACCATTGGGACGTTCGGGATCTGGGCCGCATACCTCACGGGCGGAGACACCATCTACCTGGCCAATTACACCCTCCCCGACTCCCCTTTCCTCAAAATCTTTAAGCCAGAGGCAGCCAAGG |
| 21 | ACTACACTGCTCAGGACTTCAGAATCATACTTTCTCTTGATATTTACTTATGGATGTATTTCTGTCTTTTATTTATTTCT  CTTCCGAGGCGGCGGCTCTGACGCCCAGACAGGAGTGCCATCCGGTGATATCGGCTCAACGCCGTCTCCATTTCCCTGGGTTCAAGCGATTGGCCTGGCTGACCCTCACCAGTAGTCGGGACTACTCGTGAGGGTGACCACGGCCAATCAATCTTTATACTTTTGGTGGCGGTTGGTTTCACCCGAGATCACCCCGACGGACTCCATCCCGTGACACCGAGACCCACCCCCCCCCACCACACATAATGTTGGGATGACAATACTGACCCAATGAACCCTCCCTACACTTGACGATTCTGATATCATAACCCTGCGGAAAATATTTTGACATATACAGGGATGTGTGGTGTCTTATA | ACCGCCACTTCCCGGGGGAGTACGTCCGCTTCACCGGCTACCCCTGCTCCTGGACCTTCTACCACCACCTCCGCCAGGAGATCCTCCAGGAGTTCACCCTGCACGACCACGTGCGGGAGGAGGCCCAGAAGTTCCTGCGGGGCCTGCAGGTGAACGGGAGCCGGCCGGGCACCTTTGTAGGGGTCCATGTTCGCCGAGGGGACTATGTCCATGTCATGCCAAAAGTGTGGAAGGGGGTGGTGGCCGACCGGCGATACCTACAGCAGGCCCTGGACTGGTTCCGAGCTCGCTACAGCTCCCTCATCTTCGTGGTCACCAGTAATGGCATGGCCTGGTGTCGGGAGAACATTGACACCTCCCACGGTGATGTGGTGTTTGCTGGCGATGGCATTGAGGGCTCACCTGCCAAAGATTTTGCTCTACTCACACAGTGTAACCACACCATCATGACCATTGGGACGTTCGGGATCTGGGCCGCATACCTCACGGGCGGAGACACCATCTACCTGGCCAATTACACCCTCCCCGACTCCCCTTTCCTCAAAATCTTTAAGCCAGAGGCAGCCAAGG |
| 23 | ACTACACTGCTCAGGACTTCAGAATCATACTTTCTCTTGATATTTACTTATGGATGTATTTCTGTCTTTTATTTATTTCT  CTTCCGAGGCGGCGGCTCTGACGCCCAGACAGGAGTGCCATCCGGTGATATCGGCTCAACGCCGTCTCCATTTCCCTGGGTTCAAGCGATTGGCCTGGCTGACCCTCACCAGTAGTCGGGACTACTCGTGAGGGTGACCACGGCCAATCAATCTTTATACTTTTGGTGGCGGTTGGTTTCACCCGAGATCACCCCGACGGACTCCATCCCGTGACACCGAGACCCACCCCCCCCCACCACACATAATGTTGGGATGACAATACTGACCCAATGAACCCTCCCTACACTTGACGATTCTGATATCATAACCCTGCGGAAAATATTTTGACATATACAGGGATGTGTGGTGTCTTATA | ACCGCCACTTCCCGGGGGAGTACGTCCGCTTCACCGGCTACCCCTGCTCCTGGACCTTCTACCACCACCTCCGCCAGGAGATCCTCCAGGAGTTCACCCTGCACGACCACGTGCGGGAGGAGGCCCAGAAGTTCCTGCGGGGCCTGCAGGTGAACGGGAGCCGGCCGGGCACCTTTGTAGGGGTCCATGTTCGCCGAGGGGACTATGTCCATGTCATGCCAAAAGTGTGGAAGGGGGTGGTGGCCGACCGGCGATACCTACAGCAGGCCCTGGACTGGTTCCGAGCTCGCTACAGCTCCCTCATCTTCGTGGTCACCAGTAATGGCATGGCCTGGTGTCGGGAGAACATTGACACCTCCCACGGTGATGTGGTGTTTGCTGGCGATGGCATTGAGGGCTCACCTGCCAAAGATTTTGCTCTACTCACACAGTGTAACCACACCATCATGACCATTGGGACGTTCGGGATCTGGGCCGCATACCTCACGGGCGGAGACACCATCTACCTGGCCAATTACACCCTCCCCGACTCCCCTTTCCTCAAAATCTTTAAGCCAGAGGCAGCCAAGG |
| 25 | GACATATGGTCAGGACTTCAGAATTATACTTTCTCTTGAGATGGGCTTATGGGCGCATGCCTGTCTTTTAGTTATTTCTCTTCCGAGGCGGCGGCTCTGACGCCCAGGCAGGAGTGCGTCCGGTGATCTCGGCTCAACCCCGTCTCCATTACCATGGGTTCAATTAATTGGCCTGGCTGGCCCTCACCTGTAGTTGGAACTACTCGGGACGCTCACCACGCCCAATCAATTGAACCCATTTTGGTGGCGGTTGGTTTCACCCGAGATCACCCCAACGGACTCCATCCCGTGACACCGAGACCCACCCCCCCCCACCACAAATAATGTAAAAATGACAATACTTACCCACTGAAACCTGCCTACACTTGATGATTCTGATATCATAACCCTGCGGAAAATCTTTTGACATATACACGGATGTGTGGTGTCTTATA | ACCGCCACTTCCCGGGGGAGTACGTCCGCTTCACCGGCTACCCCTGCTCCTGGACCTTCTACCACCACCTCCGCCAGGAGATCCTCCAGGAGTTCACCCTGCACGACCACGTGCGGGAGGAGGCCCAGAAGTTCCTGCGGGGCCTGCAGGTGAACGGGAGCCGGCCGGGCACCTTTGTAGGGGTCCATGTTCGCCGAGGGGACTATGTCCATGTCATGCCAAAAGTGTGGAAGGGGGTGGTGGCCGACCGGCGATACCTACAGCAGGCCCTGGACTGGTTCCGAGCTCGCTACAGCTCCCTCATCTTCGTGGTCACCAGTAATGGCATGGCCTGGTGTCGGGAGAACATTGACACCTCCCACGGTGATGTGGTGTTTGCTGGCGATGGCATTGAGGGCTCACCTGCCAAAGATTTTGCTCTACTCACACAGTGTAACCACACCATCATGACCATTGGGACGTTCGGGATCTGGGCCGCATACCTCACGGGCGGAGACACCATCTACCTGGCCAATTACACCCTCCCCGACTCCCCTTTCCTCAAAATCTTTAAGCCAGAGGCAGCCAAGG |
| 28 | TATTTGCTCAGGCCTTCAGAATTATACTTTCTCTTGATATGGACTTATGGATGTATTTCTGTTTTTTATTTATTTCTCTT  GCCGCGGCGGTCGCTCTGACGTCCAGGCTGGAGTGCGTCCGGGCCTCTCGGCTCAACCCCGTCTCCATTACCCTGATAAAAAGCGATTGGCCTGGCTGACCCTCCCCTGTAGTTGGGACTACTCGTGAGGGCCACCACGCCCAATTAATCTTTATACTTTTGGTGGCGGTTGGTTTCACCCGAGATCACCCCAACGGACTCCATCCCGTGACACCGAGACCCACCCCCCCCCACCACACATAAATTAAAAAAAATAAATACTTACCCACTGAACCCGGCCTTCTCTTGACGATTCTGATATCATAACCCTGCGGAAAATATTTTGACATATACAGGGATGTGTGGTGTCTTATA | ACCGCCACTTCCCGGGGGAGTACGTCCGCTTCACCGGCTACCCCTGCTCCTGGACCTTCTACCACCACCTCCGCCAGGAGATCCTCCAGGAGTTCACCCTGCACGACCACGTGCGGGAGGAGGCCCAGAAGTTCCTGCGGGGCCTGCAGGTGAACGGGAGCCGGCCGGGCACCTTTGTAGGGGTCCATGTTCGCCGAGGGGACTATGTCCATGTCATGCCAAAAGTGTGGAAGGGGGTGGTGGCCGACCGGCGATACCTACAGCAGGCCCTGGACTGGTTCCGAGCTCGCTACAGCTCCCTCATCTTCGTGGTCACCAGTAATGGCATGGCCTGGTGTCGGGAGAACATTGACACCTCCCACGGTGATGTGGTGTTTGCTGGCGATGGCATTGAGGGCTCACCTGCCAAAGATTTTGCTCTACTCACACAGTGTAACCACACCATCATGACCATTGGGACGTTCGGGATCTGGGCCGCATACCTCACGGGCGGAGACACCATCTACCTGGCCAATTACACCCTCCCCGACTCCCCTTTCCTCAAAATCTTTAAGCCAGAGGCAGCCAAGG |
| 31 | CAAGCACTCACTGGTTAGGACTTCAGAATCATCGTTTCTCTTGATATTGACTTATGGGCGTATTCCTGTCTTCCCATTTATTTCTCTTGCCGAGGCAGGCGGCTCTGTCGTCCAGAGAGGAGTGCCATCCGGGCCGATCGGCTCAACCCCGTCTCCATTACAATGGGTTCAATTAATTGGCCTGGCTGACCCTCACCAGTAGTTGGGACTACTCGGGACGCTCACCACGCCCAATCAATCTTTATACTTTTGGTGGCGGTTGGTTGACCCGAGATCACCCCGACGGACTCCATCCCGTGACACCGAGCCCCACCCCCCCCCGCCACACATAAATTTGGGAAATAAATACTGACCCACTGAACCCGGCCTACTCTTGACGATTCTGATATCATAACCCTGTGGAAAATCTTTTGACATATACAGGGATGTGTGGTGTCGTGT | ACCGCCACTTCCCGGGGGAGTACGTCCGCTTCACCGGCTACCCCTGCTCCTGGACCTTCTACCACCACCTCCGCCAGGAGATCCTCCAGGAGTTCACCCTGCACGACCACGTGCGGGAGGAGGCCCAGAAGTTCCTGCGGGGCCTGCAGGTGAACGGGAGCCGGCCGGGCACCTTTGTAGGGGTCCATGTTCGCCGAGGGGACTATGTCCATGTCATGCCAAAAGTGTGGAAGGGGGTGGTGGCCGACCGGCGATACCTACAGCAGGCCCTGGACTGGTTCCGAGCTCGCTACAGCTCCCTCATCTTCGTGGTCACCAGTAATGGCATGGCCTGGTGTCGGGAGAACATTGACACCTCCCACGGTGATGTGGTGTTTGCTGGCGATGGCATTGAGGGCTCACCTGCCAAAGATTTTGCTCTACTCACACAGTGTAACCACACCATCATGACCATTGGGACGTTCGGGATCTGGGCCGCATACCTCACGGGCGGAGACACCATCTACCTGGCCAATTACACCCTCCCCGACTCCCCTTTCCTCAAAATCTTTAAGCCAGAGGCAGCCAAGG |
| 35 | TATTTGCTCAGGCCTTCAGAATTATACTTTCTCTTGATATGGACTTATGGATGTATTTCTGTTTTTTATTTATTTCTCTT  GCCGCGGCGGTCGCTCTGACGTCCAGGCTGGAGTGCGTCCGGGCCTCTCGGCTCAACCCCGTCTCCATTACCCTGATAAAAAGCGATTGGCCTGGCTGACCCTCCCCTGTAGTTGGGACTACTCGTGAGGGCCACCACGCCCAATTAATCTTTATACTTTTGGTGGCGGTTGGTTTCACCCGAGATCACCCCAACGGACTCCATCCCGTGACACCGAGACCCACCCCCCCCCACCACACATAAATTAAAAAAAATAAATACTTACCCACTGAACCCGGCCTTCTCTTGACGATTCTGATATCATAACCCTGCGGAAAATATTTTGACATATACAGGGATGTGTGGTGTCTTATA | ACCGCCACTTCCCGGGGGAGTACGTCCGCTTCACCGGCTACCCCTGCTCCTGGACCTTCTACCACCACCTCCGCCAGGAGATCCTCCAGGAGTTCACCCTGCACGACCACGTGCGGGAGGAGGCCCAGAAGTTCCTGCGGGGCCTGCAGGTGAACGGGAGCCGGCCGGGCACCTTTGTAGGGGTCCATGTTCGCCGAGGGGACTATGTCCATGTCATGCCAAAAGTGTGGAAGGGGGTGGTGGCCGACCGGCGATACCTACAGCAGGCCCTGGACTGGTTCCGAGCTCGCTACAGCTCCCTCATCTTCGTGGTCACCAGTAATGGCATGGCCTGGTGTCGGGAGAACATTGACACCTCCCACGGTGATGTGGTGTTTGCTGGCGATGGCATTGAGGGCTCACCTGCCAAAGATTTTGCTCTACTCACACAGTGTAACCACACCATCATGACCATTGGGACGTTCGGGATCTGGGCCGCATACCTCACGGGCGGAGACACCATCTACCTGGCCAATTACACCCTCCCCGACTCCCCTTTCCTCAAAATCTTTAAGCCAGAGGCAGCCAAGG |
| 40 | GACATATGGTCAGGACTTCAGAATTATACTTTCTCTTGAGATGGGCTTATGGGCGCATGCCTGTCTTTTAGTTATTTCTCTTCCGAGGCGGCGGCTCTGACGCCCAGGCAGGAGTGCGTCCGGTGATCTCGGCTCAACCCCGTCTCCATTACCATGGGTTCAATTAATTGGCCTGGCTGGCCCTCACCTGTAGTTGGAACTACTCGGGACGCTCACCACGCCCAATCAATTGAACCCATTTTGGTGGCGGTTGGTTTCACCCGAGATCACCCCAACGGACTCCATCCCGTGACACCGAGACCCACCCCCCCCCACCACAAATAATGTAAAAATGACAATACTTACCCACTGAAACCTGCCTACACTTGATGATTCTGATATCATAACCCTGCGGAAAATCTTTTGACATATACACGGATGTGTGGTGTCTTATA | ACCGCCACTTCCCGGGGGAGTACGTCCGCTTCACCGGCTACCCCTGCTCCTGGACCTTCTACCACCACCTCCGCCAGGAGATCCTCCAGGAGTTCACCCTGCACGACCACGTGCGGGAGGAGGCCCAGAAGTTCCTGCGGGGCCTGCAGGTGAACGGGAGCCGGCCGGGCACCTTTGTAGGGGTCCATGTTCGCCGAGGGGACTATGTCCATGTCATGCCAAAAGTGTGGAAGGGGGTGGTGGCCGACCGGCGATACCTACAGCAGGCCCTGGACTGGTTCCGAGCTCGCTACAGCTCCCTCATCTTCGTGGTCACCAGTAATGGCATGGCCTGGTGTCGGGAGAACATTGACACCTCCCACGGTGATGTGGTGTTTGCTGGCGATGGCATTGAGGGCTCACCTGCCAAAGATTTTGCTCTACTCACACAGTGTAACCACACCATCATGACCATTGGGACGTTCGGGATCTGGGCCGCATACCTCACGGGCGGAGACACCATCTACCTGGCCAATTACACCCTCCCCGACTCCCCTTTCCTCAAAATCTTTAAGCCAGAGGCAGCCAAGG |
| 41 | TATTTGCTCAGGCCTTCAGAATTATACTTTCTCTTGATATGGACTTATGGATGTATTTCTGTTTTTTATTTATTTCTCTT  GCCGCGGCGGTCGCTCTGACGTCCAGGCTGGAGTGCGTCCGGGCCTCTCGGCTCAACCCCGTCTCCATTACCCTGATAAAAAGCGATTGGCCTGGCTGACCCTCCCCTGTAGTTGGGACTACTCGTGAGGGCCACCACGCCCAATTAATCTTTATACTTTTGGTGGCGGTTGGTTTCACCCGAGATCACCCCAACGGACTCCATCCCGTGACACCGAGACCCACCCCCCCCCACCACACATAAATTAAAAAAAATAAATACTTACCCACTGAACCCGGCCTTCTCTTGACGATTCTGATATCATAACCCTGCGGAAAATATTTTGACATATACAGGGATGTGTGGTGTCTTATA | ACCGCCACTTCCCGGGGGAGTACGTCCGCTTCACCGGCTACCCCTGCTCCTGGACCTTCTACCACCACCTCCGCCAGGAGATCCTCCAGGAGTTCACCCTGCACGACCACGTGCGGGAGGAGGCCCAGAAGTTCCTGCGGGGCCTGCAGGTGAACGGGAGCCGGCCGGGCACCTTTGTAGGGGTCCATGTTCGCCGAGGGGACTATGTCCATGTCATGCCAAAAGTGTGGAAGGGGGTGGTGGCCGACCGGCGATACCTACAGCAGGCCCTGGACTGGTTCCGAGCTCGCTACAGCTCCCTCATCTTCGTGGTCACCAGTAATGGCATGGCCTGGTGTCGGGAGAACATTGACACCTCCCACGGTGATGTGGTGTTTGCTGGCGATGGCATTGAGGGCTCACCTGCCAAAGATTTTGCTCTACTCACACAGTGTAACCACACCATCATGACCATTGGGACGTTCGGGATCTGGGCCGCATACCTCACGGGCGGAGACACCATCTACCTGGCCAATTACACCCTCCCCGACTCCCCTTTCCTCAAAATCTTTAAGCCAGAGGCAGCCAAGG |
| 52 | CAAGCTCTTACTGGTCAGAACTTCAGAATTATACGTTTCTCTTGATATTTACTTATGGGCTCATTTCTGTTTTCCCATTTATTTCTCTTGAGAGGCGTCGGCTCTGACGTCCAGACAGGAGTGCCATCCGGTGATCTCGGCTCAACGCCGCCGCCACCTCCCTGGGTTCAAGCGATTGGCCTGGCTGACCCTCCCCAGTAGTTGGAACTACTCGTGACGCTCACCACGCCCAATCAATCTTTATACTTTTGGTGGCGGTTGGTTTCACCCGAGATCACCCCGACGGACTCCATCCCGTGACACCGAGACCCACCCCCCCCCGCCACACATAATGTAAAAATGACAATACTGACCCACTGAACCCTGCCTACACTTGACGATTCTGATATCATAACCCTGCGGAAAATATTTTGACATATACACGGAGATGTGGTGTCTTATA | ACCGCCACTTCCCGGGGGAGTACGTCCGCTTCACCGGCTACCCCTGCTCCTGGACCTTCTACCACCACCTCCGCCAGGAGATCCTCCAGGAGTTCACCCTGCACGACCACGTGCGGGAGGAGGCCCAGAAGTTCCTGCGGGGCCTGCAGGTGAACGGGAGCCGGCCGGGCACCTTTGTAGGGGTCCATGTTCGCCGAGGGGACTATGTCCATGTCATGCCAAAAGTGTGGAAGGGGGTGGTGGCCGACCGGCGATACCTACAGCAGGCCCTGGACTGGTTCCGAGCTCGCTACAGCTCCCTCATCTTCGTGGTCACCAGTAATGGCATGGCCTGGTGTCGGGAGAACATTGACACCTCCCACGGTGATGTGGTGTTTGCTGGCGATGGCATTGAGGGCTCACCTGCCAAAGATTTTGCTCTACTCACACAGTGTAACCACACCATCATGACCATTGGGACGTTCGGGATCTGGGCCGCATACCTCACGGGCGGAGACACCATCTACCTGGCCAATTACACCCTCCCCGACTCCCCTTTCCTCAAAATCTTTAAGCCAGAGGCAGCCAAGG |
