# Supplementary material for: Fucosylated Human Milk Oligosaccharides and N-Glycans in the Milk of Chinese Mothers Regulate the Gut Microbiome of Their Breast-Fed Infants during Different Lactation Stages
Source: mSystems. 2018 Dec 26;3(6):e00206-18. doi: 10.1128/mSystems.00206-18 (PMC6306508; doi:10.1128/mSystems.00206-18)
Supplement: TABLE S2 [file sys006182308st2.docx]

TABLE S2. The major HMOs fractions in breast milk detected by LC-MS

| HMO fractions | Retention Time (min) | m/z |
| --- | --- | --- |
| H2F1 | 11.3 | 487-488 |
| H3 | 12.6 | 503-504 |
| H2F2 | 14.3 | 679-680 |
| H3N1 | 16.0 | 706-707 |
| H3N1F1 | 19.0 | 852-853 |
| H3N1F2 | 21.9 | 998-999 |
| H4N2 | 21.5 | 1072-1073 |
| H4N2F1 | 23.6 | 1217-1218 |
| H4N2F2 | 26.2 | 1363-1364 |
| H4N2F3 | 28.1 | 777-779 |
| H5N3F1 | 27.3 | 790-792 |
| H5N3F2 | 29 | 863-865 |
| H5N3F3 | 30.5 | 936-938 |
| H6N4F1 | 30 | 972-974 |
| H5N3F4 | 31.9 | 1009-1011 |
| H6N4F2 | 31.4 | 1046-1048 |
| H6N4F3 | 32.6 | 1119-1121 |
| H2A1 | 2.9 | 632-633 |
| H1F1A1 | 3.8 | 673-674 |
| H3N1A1 | 2.9 | 997-998 |
| H3N1F1A1 | 3.8 | 1143-1144 |
| H3N1A2 | 2.9 | 1288-1289 |
| H4N2A1 | 5.0 | 1362-1363 |
| H4N2F1A1 | 6.0 | 1508-1509 |
| H4N2A2 | 3.4 | 826-827 |
| H4N2F2A1 | 7.3 | 827-828 |
| H4N2F1A2 | 3.4 | 899-901 |
| H5N3A1 | 7.3 | 863-865 |
| H5N3F3A1 | 3.4 | 1082-1084 |
| H6N4F1A1 | 7.4 | 1118-1120 |
