# Supplementary figures and images for: Fucosylated Human Milk Oligosaccharides and N-Glycans in the Milk of Chinese Mothers Regulate the Gut Microbiome of Their Breast-Fed Infants during Different Lactation Stages
Source: mSystems. 2018 Dec 26;3(6):e00206-18. doi: 10.1128/mSystems.00206-18 (PMC6306508; doi:10.1128/mSystems.00206-18)

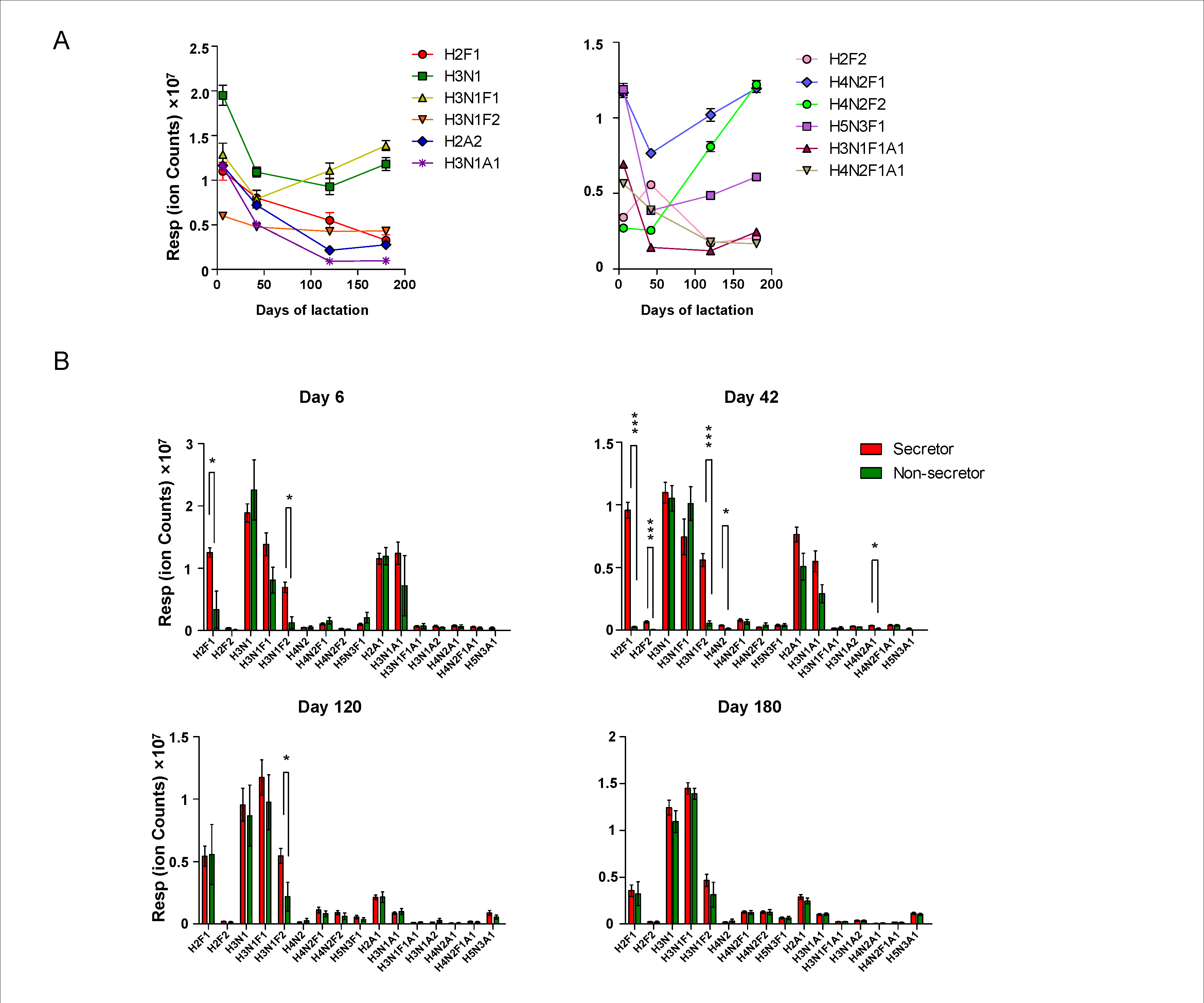

Supplement: FIG S1 [file sys006182308sf1.tif]

A

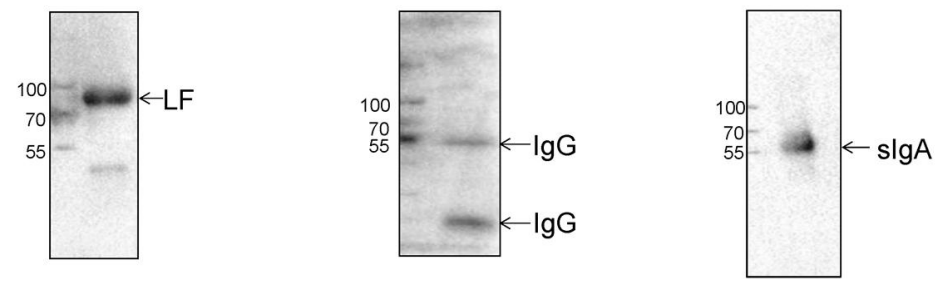

B

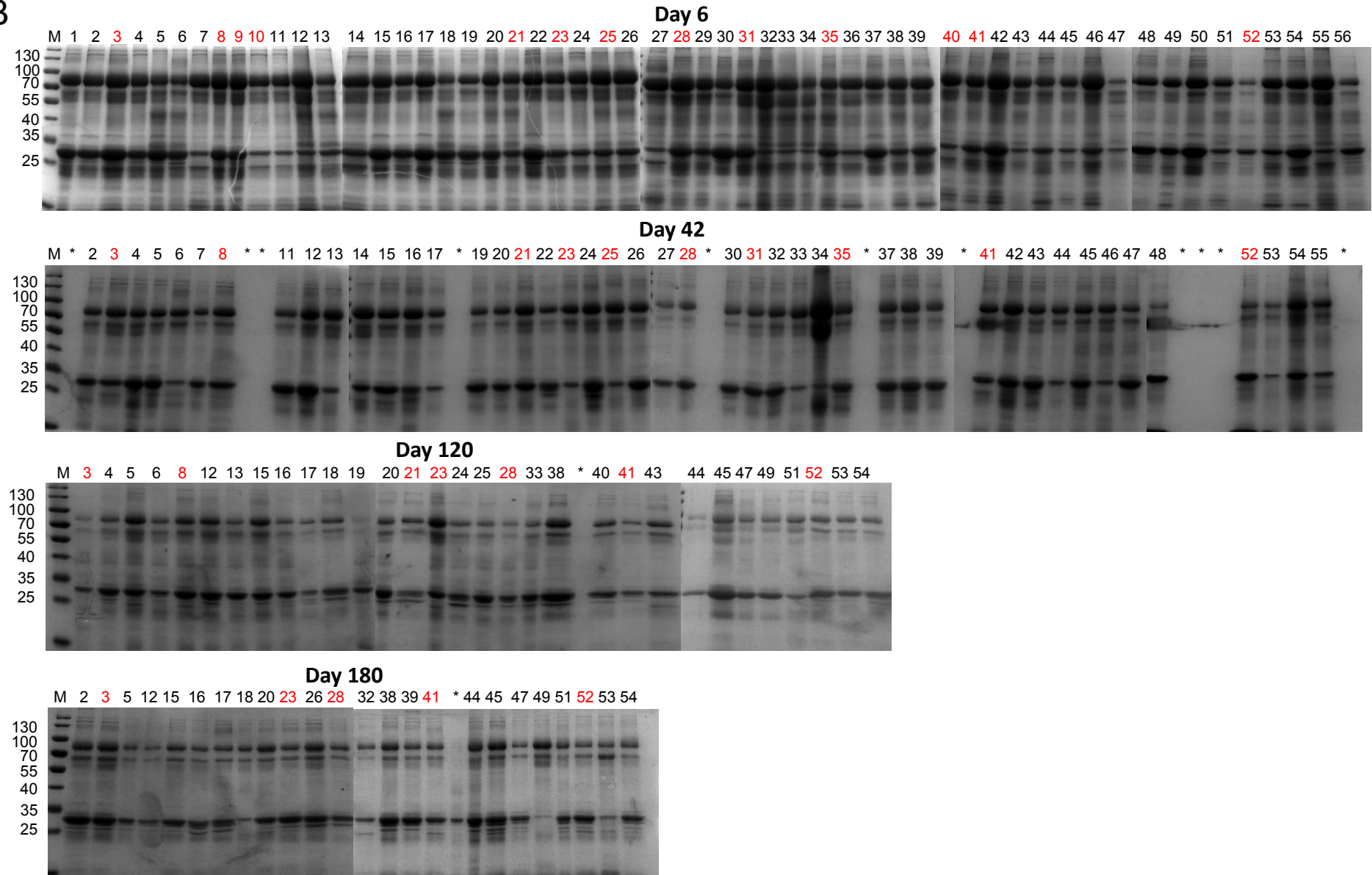

Supplement: FIG S2 [file sys006182308sf2.pdf]

A

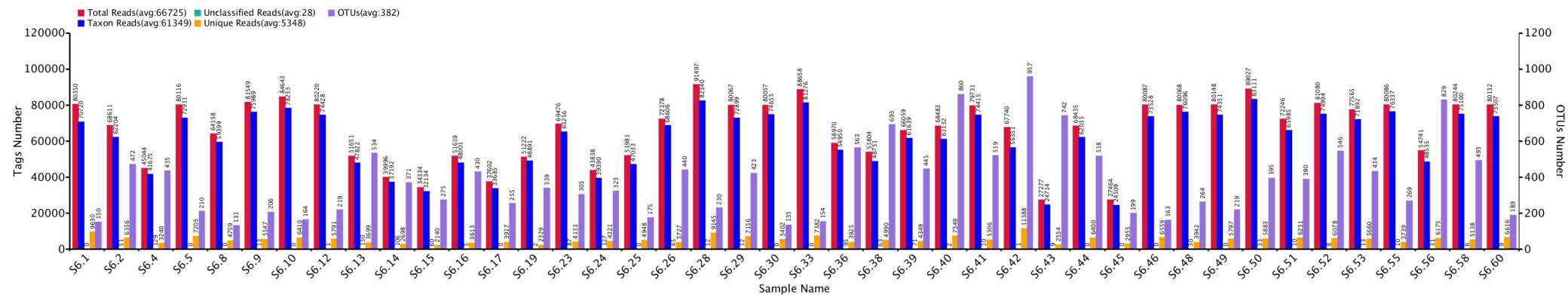

B

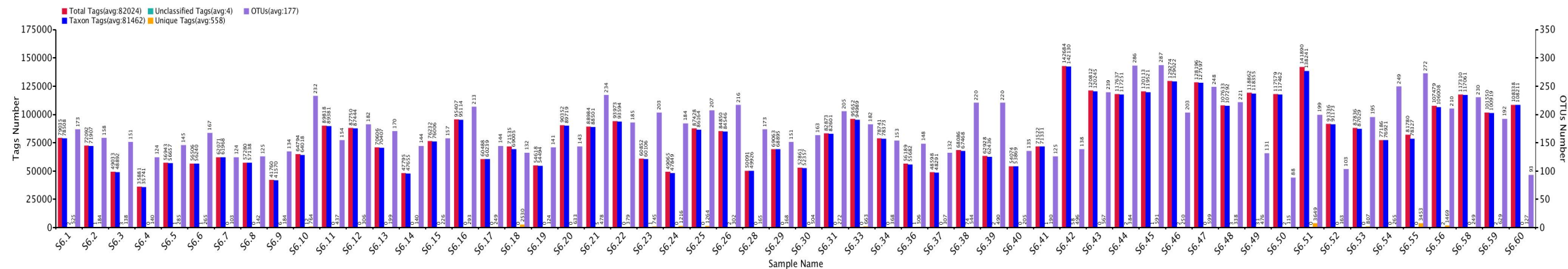

C

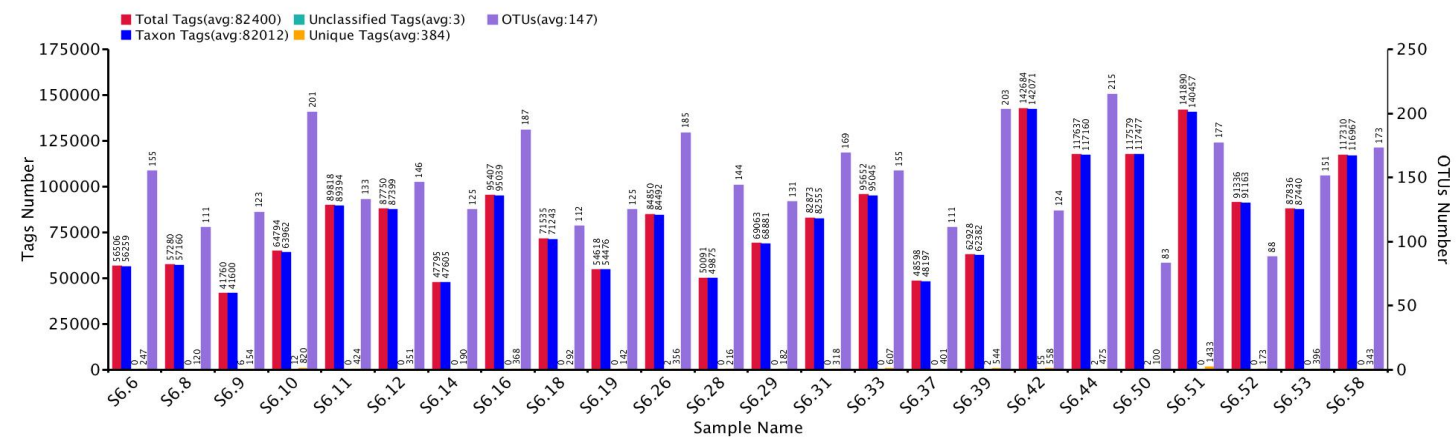

Supplement: FIG S3 [file sys006182308sf3.pdf]

A

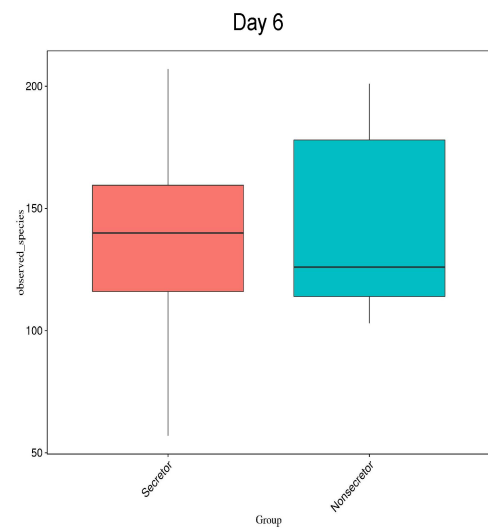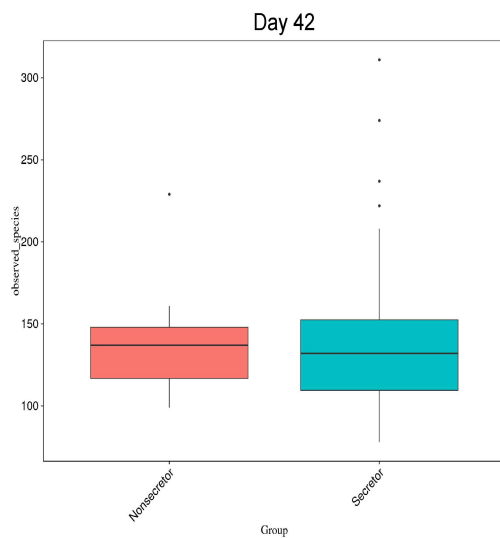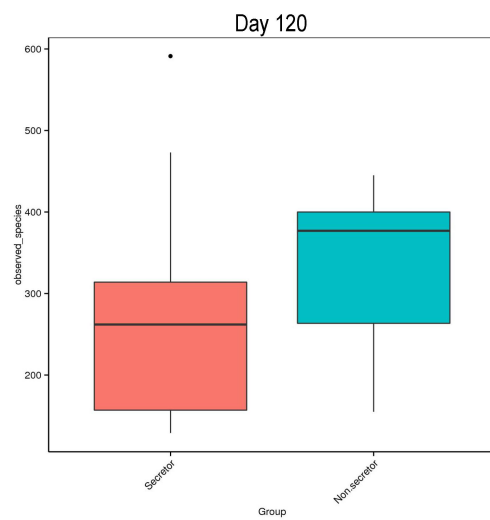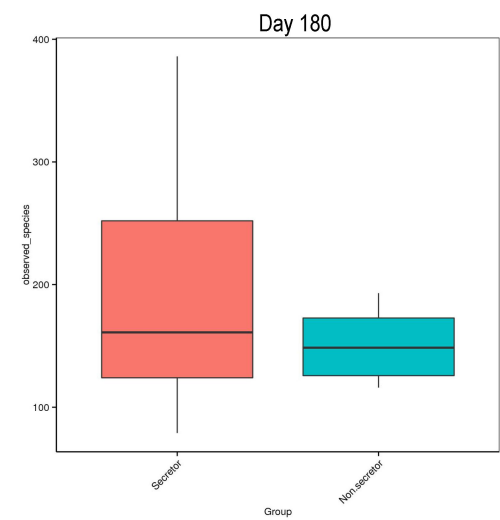

B

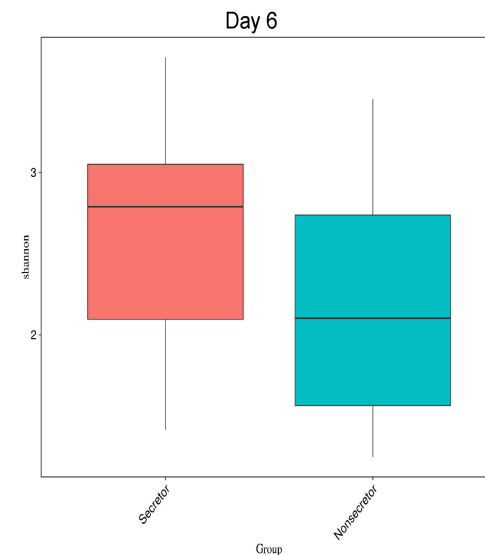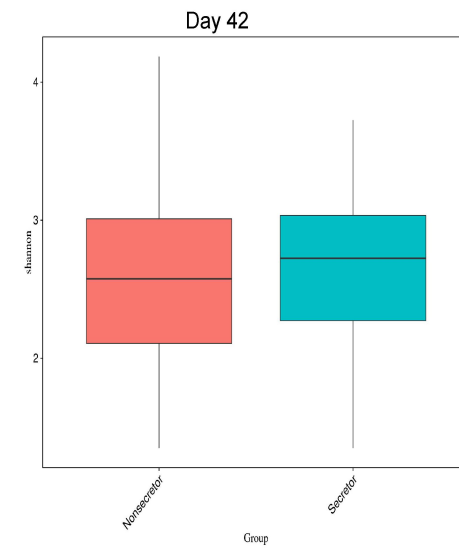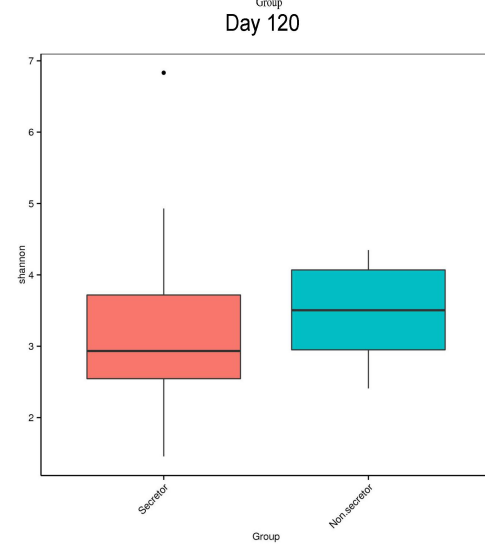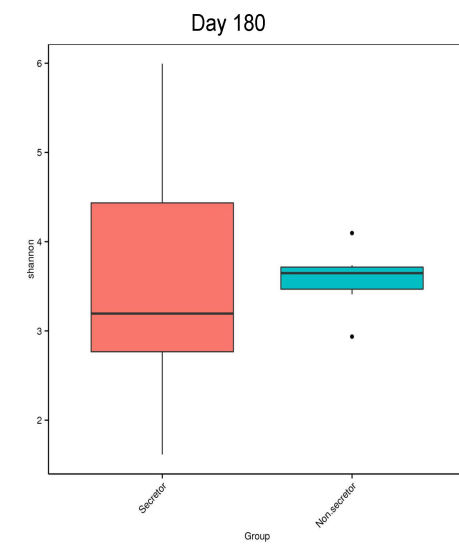

Supplement: FIG S4 [file sys006182308sf4.pdf]

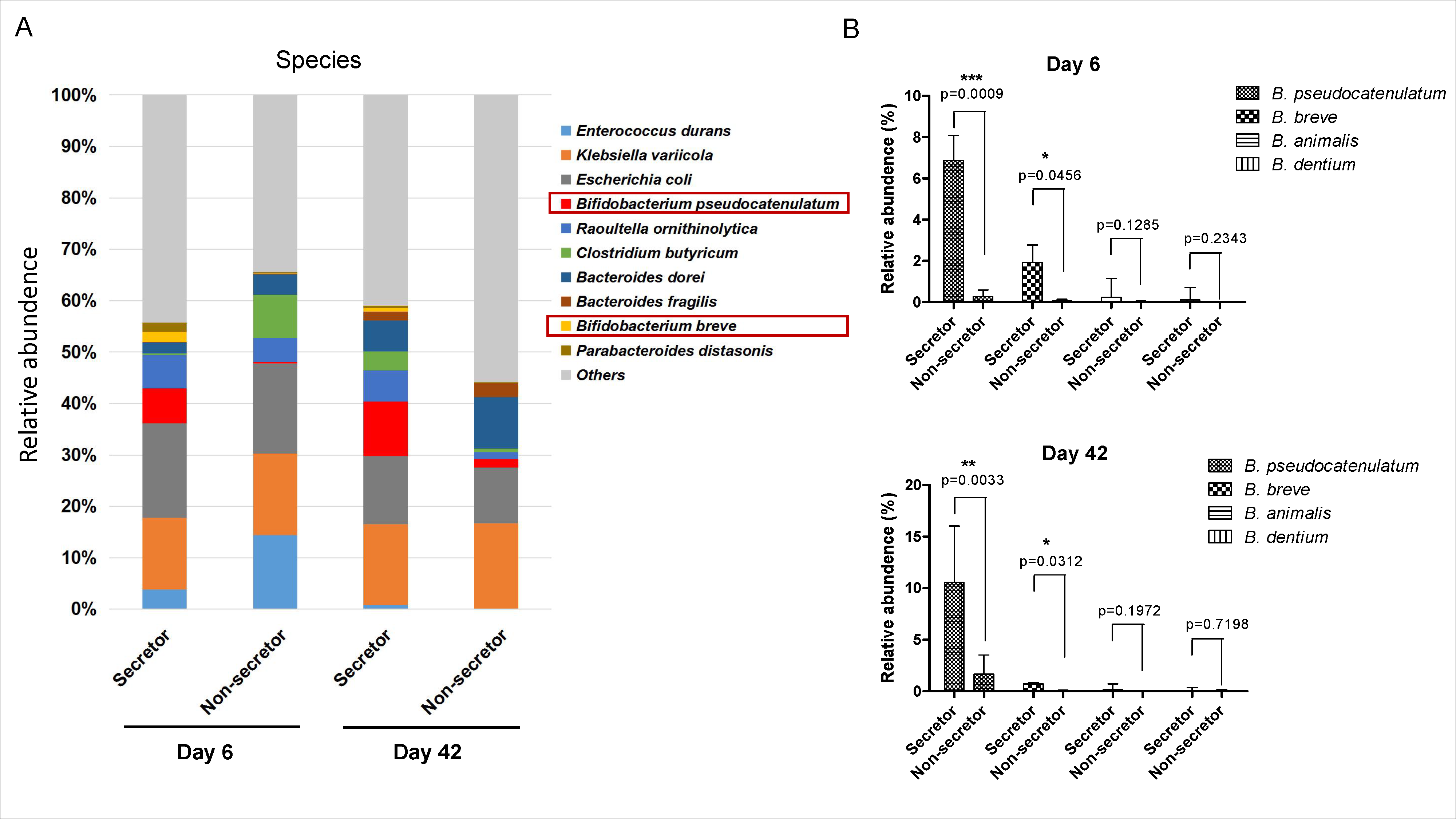

Supplement: FIG S5 [file sys006182308sf5.tif]

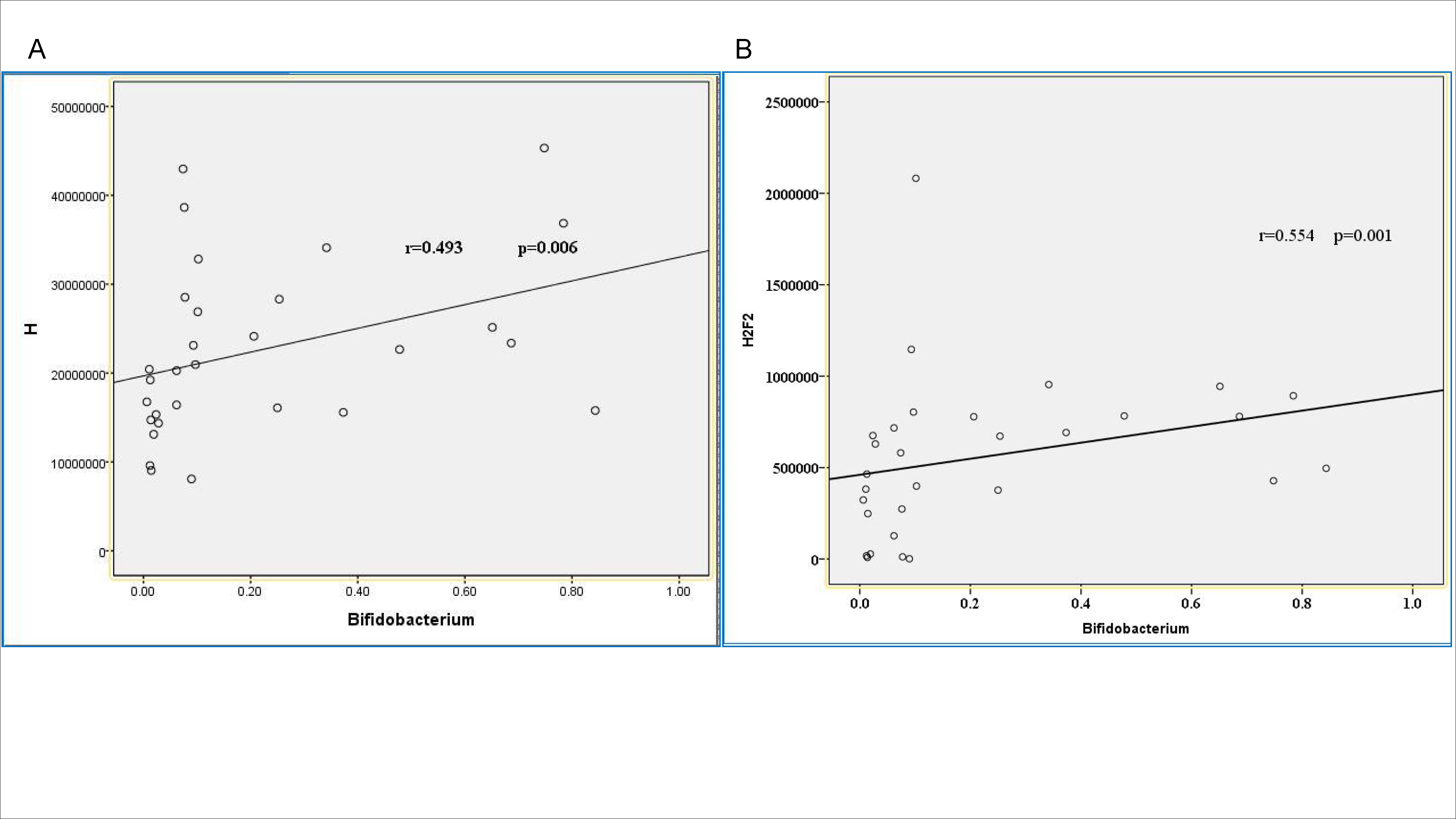

Supplement: FIG S6 [file sys006182308sf6.tif]
