# Supplementary material for: Fucosylated Human Milk Oligosaccharides and N-Glycans in the Milk of Chinese Mothers Regulate the Gut Microbiome of Their Breast-Fed Infants during Different Lactation Stages
Source: mSystems. 2018 Dec 26;3(6):e00206-18. doi: 10.1128/mSystems.00206-18 (PMC6306508; doi:10.1128/mSystems.00206-18)
Supplement: TABLE S3 [file sys006182308st3.docx]

TABLE S3. The total tags and OTU numbers derived from 16S rDNA sequencing of fecal samples of infants collected by day 120 post birth.

| Sample_Name | Total_tag | Taxon_Tag | Unclassified_Tag | Unique_Tag | OTU_num |
| --- | --- | --- | --- | --- | --- |
| S42.2 | 228589 | 228253 | 0 | 336 | 215 |
| S42.4 | 163576 | 162505 | 0 | 1071 | 207 |
| S42.5 | 40954 | 40872 | 0 | 82 | 162 |
| S42.6 | 69473 | 69342 | 0 | 131 | 217 |
| S42.8 | 147386 | 147162 | 0 | 224 | 209 |
| S42.9 | 92120 | 91966 | 0 | 154 | 218 |
| S42.10 | 101478 | 101317 | 0 | 161 | 181 |
| S42.11 | 60086 | 59887 | 0 | 199 | 152 |
| S42.12 | 56158 | 56015 | 0 | 143 | 181 |
| S42.13 | 67047 | 66868 | 0 | 179 | 193 |
| S42.14 | 125648 | 125405 | 0 | 243 | 212 |
| S42.15 | 81617 | 81459 | 0 | 158 | 190 |
| S42.16 | 75358 | 75077 | 0 | 281 | 208 |
| S42.17 | 40743 | 40557 | 0 | 186 | 237 |
| S42.18 | 102894 | 102644 | 29 | 221 | 272 |
| S42.19 | 101230 | 100763 | 0 | 467 | 354 |
| S42.20 | 81870 | 81602 | 0 | 268 | 274 |
| S42.21 | 104781 | 104620 | 0 | 161 | 204 |
| S42.24 | 80958 | 80504 | 0 | 454 | 318 |
| S42.25 | 107885 | 107416 | 0 | 469 | 267 |
| S42.26 | 120672 | 120367 | 0 | 305 | 284 |
| S42.27 | 115840 | 115685 | 0 | 155 | 149 |
| S42.28 | 78102 | 78022 | 0 | 80 | 137 |
| S42.29 | 78669 | 78594 | 0 | 75 | 150 |
| S42.30 | 116577 | 116353 | 0 | 224 | 193 |
| S42.31 | 104875 | 104781 | 0 | 94 | 202 |
| S42.32 | 112506 | 111924 | 0 | 582 | 157 |
| S42.33 | 91877 | 91637 | 0 | 240 | 147 |
| S42.34 | 76788 | 76717 | 0 | 71 | 150 |
| S42.37 | 80774 | 80410 | 0 | 364 | 159 |
| S42.38 | 144729 | 144287 | 0 | 442 | 210 |
| S42.39 | 134176 | 133945 | 0 | 231 | 136 |
| S42.41 | 98090 | 97983 | 0 | 107 | 134 |
| S42.42 | 92052 | 91876 | 0 | 176 | 179 |
| S42.43 | 91084 | 90999 | 0 | 85 | 197 |
| S42.44 | 127784 | 127612 | 0 | 172 | 155 |
| S42.45 | 106344 | 106247 | 0 | 97 | 132 |
| S42.46 | 115619 | 114772 | 0 | 847 | 158 |
| S42.47 | 106075 | 105938 | 0 | 137 | 169 |
| S42.48 | 165610 | 165367 | 0 | 243 | 226 |
| S42.49 | 124586 | 124383 | 0 | 203 | 167 |
| S42.54 | 141695 | 140824 | 0 | 871 | 227 |
| S42.55 | 126790 | 124456 | 0 | 2334 | 221 |
| S42.56 | 144363 | 143665 | 6 | 692 | 189 |
| S42.57 | 123616 | 121700 | 0 | 1916 | 151 |
| S42.58 | 108829 | 108667 | 0 | 162 | 171 |
| S42.59 | 141789 | 141599 | 4 | 186 | 184 |
| S42.40 | 100146 | 100068 | 0 | 78 | 111 |
| S42.50 | 98632 | 98462 | 0 | 170 | 159 |
