# Supplementary material for: Fucosylated Human Milk Oligosaccharides and N-Glycans in the Milk of Chinese Mothers Regulate the Gut Microbiome of Their Breast-Fed Infants during Different Lactation Stages
Source: mSystems. 2018 Dec 26;3(6):e00206-18. doi: 10.1128/mSystems.00206-18 (PMC6306508; doi:10.1128/mSystems.00206-18)
Supplement: TABLE S4 [file sys006182308st4.docx]

TABLE S4. The statistic test of beta diversity of gut microbiota between infants fed by secretor and non-secretor mothers.

| Secretor vs. Non-secretor | | | | |
| --- | --- | --- | --- | --- |
| Test / p value | Day 6 | Day 42 | Day 120 | Day 180 |
| unweighted_unifrac_t-test | 0.1135 | 1.38E-06 | 0.7991 | 0.3736 |
| unweighted_unifrac_two-wilcox | 0.1587 | 1.06E-05 | 0.7638 | 0.3807 |
| weighted_unifrac_t-test | 0.1438 | 0.0015 | 0.3529 | 0.2808 |
| weighted_unifrac_two-wilcox | 0.0864 | 0.0056 | 0.2637 | 0.2313 |
